# Supplementary material for: Evaluation of a city-wide school-located influenza vaccination program in Oakland, California, with respect to vaccination coverage, school absences, and laboratory-confirmed influenza: A matched cohort study
Source: PLoS Med. 2020 Aug 18;17(8):e1003238. doi: 10.1371/journal.pmed.1003238 (PMC7433855; doi:10.1371/journal.pmed.1003238)
Supplement: S1 Checklist — (DOCX) [file pmed.1003238.s001.docx]

STROBE Statement—Checklist of items that should be included in reports of ***cohort studies***

|  | | Item No | Recommendation | Page No |
| --- | --- | --- | --- | --- |
| **Title and abstract** | | 1 | (*a*) Indicate the study’s design with a commonly used term in the title or the abstract |  |
|  |  |  | (*b*) Provide in the abstract an informative and balanced summary of what was done and what was found | Title, Abstract paragraphs 2-4 |
| Introduction | | | | |
| Background/rationale | | 2 | Explain the scientific background and rationale for the investigation being reported | Introduction paragraphs 1-3 |
| Objectives | | 3 | State specific objectives, including any prespecified hypotheses | Introduction paragraph 4 |
| Methods | | | | |
| Study design | | 4 | Present key elements of study design early in the paper | Methods paragraphs 1, 3-6 |
| Setting | | 5 | Describe the setting, locations, and relevant dates, including periods of recruitment, exposure, follow-up, and data collection | Methods paragraphs 1-2, 7-9 |
| Participants | | 6 | (*a*) Give the eligibility criteria, and the sources and methods of selection of participants. Describe methods of follow-up | Methods paragraphs 1, 3-6 |
|  |  |  | (*b*) For matched studies, give matching criteria and number of exposed and unexposed |  |
| Variables | | 7 | Clearly define all outcomes, exposures, predictors, potential confounders, and effect modifiers. Give diagnostic criteria, if applicable | Methods paragraphs 7-9, 11 |
| Data sources/ measurement | | 8* | For each variable of interest, give sources of data and details of methods of assessment (measurement). Describe comparability of assessment methods if there is more than one group | Methods paragraphs 5, 7-9 |
| Bias | | 9 | Describe any efforts to address potential sources of bias | Methods paragraphs 15-16 |
| Study size | | 10 | Explain how the study size was arrived at | Methods paragraph 9, S3 Appendix |
| Quantitative variables | | 11 | Explain how quantitative variables were handled in the analyses. If applicable, describe which groupings were chosen and why | Methods paragraphs 11-14 |
| Statistical methods | | 12 | (*a*) Describe all statistical methods, including those used to control for confounding |  |
|  |  |  | (*b*) Describe any methods used to examine subgroups and interactions |  |
|  |  |  | (*c*) Explain how missing data were addressed | Methods paragraphs 11-17 |
|  |  |  | (*d*) If applicable, explain how loss to follow-up was addressed |  |
|  |  |  | (*e*) Describe any sensitivity analyses |  |
| Results | | | |  |
| Participants | | 13* | (a) Report numbers of individuals at each stage of study—eg numbers potentially eligible, examined for eligibility, confirmed eligible, included in the study, completing follow-up, and analysed | Results paragraph 2, S8 Appendix |
|  |  |  | (b) Give reasons for non-participation at each stage |  |
|  |  |  | (c) Consider use of a flow diagram |  |
| Descriptive data | | 14* | (a) Give characteristics of study participants (eg demographic, clinical, social) and information on exposures and potential confounders | Results paragraph 1, Table 1 |
|  |  |  | (b) Indicate number of participants with missing data for each variable of interest |  |
|  |  |  | (c) Summarise follow-up time (eg, average and total amount) |  |
| Outcome data | | 15* | Report numbers of outcome events or summary measures over time | Results paragraph 3-6, S9 Appendix Table 3 and 4 |
| Main results | 16 | (*a*) Give unadjusted estimates and, if applicable, confounder-adjusted estimates and their precision (eg, 95% confidence interval). Make clear which confounders were adjusted for and why they were included | |  |
|  |  | (*b*) Report category boundaries when continuous variables were categorized | |  |
|  |  | (*c*) If relevant, consider translating estimates of relative risk into absolute risk for a meaningful time period | | Results paragraph 3-6, Table 2, S9 Appendix Table 3 and 4 |
| Other analyses | 17 | Report other analyses done—eg analyses of subgroups and interactions, and sensitivity analyses | | Results paragraph 7 |
| Discussion | | | | |
| Key results | 18 | Summarise key results with reference to study objectives | | Discussion paragraph 1 |
| Limitations | 19 | Discuss limitations of the study, taking into account sources of potential bias or imprecision. Discuss both direction and magnitude of any potential bias | | Discussion paragraphs 5-8 |
| Interpretation | 20 | Give a cautious overall interpretation of results considering objectives, limitations, multiplicity of analyses, results from similar studies, and other relevant evidence | | Discussion paragraphs 2-4 |
| Generalisability | 21 | Discuss the generalisability (external validity) of the study results | | Discussion paragraphs 3-4 |
| Other information | | | | |
| Funding | 22 | Give the source of funding and the role of the funders for the present study and, if applicable, for the original study on which the present article is based | | Acknowledgements |

*Give information separately for exposed and unexposed groups.

**Note:** An Explanation and Elaboration article discusses each checklist item and gives methodological background and published examples of transparent reporting. The STROBE checklist is best used in conjunction with this article (freely available on the Web sites of PLoS Medicine at http://www.plosmedicine.org/, Annals of Internal Medicine at http://www.annals.org/, and Epidemiology at http://www.epidem.com/). Information on the STROBE Initiative is available at http://www.strobe-statement.org.
