# Supplementary material for: Evaluation of a city-wide school-located influenza vaccination program in Oakland, California, with respect to vaccination coverage, school absences, and laboratory-confirmed influenza: A matched cohort study
Source: PLoS Med. 2020 Aug 18;17(8):e1003238. doi: 10.1371/journal.pmed.1003238 (PMC7433855; doi:10.1371/journal.pmed.1003238)
Supplement: S1 Appendix — (PDF) [file pmed.1003238.s002.pdf]

Appendix to *Evaluation of a city-wide school-located influenza vaccination program in Oakland, California with respect to vaccination coverage, school absences, and laboratory-confirmed influenza: a matched cohort study*

S1 Appendix. Selection of schools for the vaccine coverage survey

Matching of school pairs

We used a two-stage matching algorithm to select intervention and control schools in order to account for co-located schools (i.e., schools at the same location), which are likely to have highly correlated transmission dynamics and cannot be treated as independent units. In the first stage, we used the Genetic multivariate matching algorithm [40] to match all K-5 or K-6 non-charter, public schools in each district. We treated co-located schools as independent units during this step. For any co-located schools for which both schools were selected, we chose to keep the school with the smaller generalized Mahalanobis distance to its pair. We excluded the other school in the pair from the matching process. If only one school in a co-located pair matched in the first stage, we dropped its pair from the second stage. In the second stage, we repeated the matching process again following the removal of co-located school pairs, identifying matched 34 school-pairs.

Selection of 22 school pairs for the vaccine coverage survey

We selected schools for the vaccine coverage survey using two metrics: 1) similarity of schools within pairs and 2) representativeness of district-level characteristics. To measure the similarity of schools within pairs, we calculated the generalized Mahalanobis distance between each pair of schools. To assess representativeness, we calculated the percent difference in the mean/percentage pre-intervention school-level characteristics from the California Department of Education for each school vs. the mean across all schools in a given district (N=50 elementary schools in Oakland, N=34 schools in West Contra Costa). We then took the sum of the absolute value of differences for each pair of schools. To assess dissimilarity between each school and its district, we calculated the mean of the absolute value of the sum of differences. Schools with the smallest value of dissimilarity were the most similar to their district as a whole. We chose the 22 schools with the smallest dissimilarity and distance values. One school pair was excluded from consideration by the recommendation of the district due to concerns about potential poor response rates (Stege Elementary / Lafayette Elementary).

Table A. Pre-intervention school-level characteristics in the program and comparison site

	All schools			Matched schools		
**Characteristic**	**Comparison**	**Intervention**	**Standardized difference**	**Comparison**	**Intervention**	**Standardized difference**
Mean enrollment	478.9	380.0	-35.3	478.9	391.1	-30.5
Mean class size	26.1	23.2	-18.1	26.1	23.0	-19.2
Race (%)						
Asian	10.5	10.6	1.7	10.5	11.2	7.6
Pacific Islander	0.9	1.1	24.7	0.9	1.2	28.4
Filipino	4.7	0.7	-93.7	4.7	0.8	-91.1
Hispanic	51.7	40.7	-32.5	51.7	38.8	-39.6
African American	18.5	29.6	50.1	18.5	30.3	52.3
White	12.3	12.3	-0.2	12.3	12.6	2.2
Mixed Race	1.1	3.3	84.1	1.1	3.5	87.7
Parents’ highest education level (%)						
Less than high school	24.9	26.5	6.6	24.9	26.2	5.6
High school	36.6	24.3	-63.8	36.6	23.0	-73.4
Some college	16.9	20.7	24.5	16.9	20.9	25.5

Bachelor’s degree	15.6	15.2	-2.7	15.6	15.8	1.0
Graduate degree	6.0	13.4	65.2	6.0	14.2	69.3
% English language learners	42.8	37.4	-17.9	42.8	35.6	-25.1
% Receiving free or reduced price lunch	70.9	74.0	5.5	70.9	72.0	2.0
Mean API score 2013	761.3	769.9	1.6	761.3	771.1	1.8
Mean API score 2012	772.3	792.0	3.5	772.3	794.2	3.9
Mean CST score - English	349.1	352.0	1.2	349.1	353.4	1.7
% Advanced CST score - English	21.3	23.1	9.3	21.3	23.9	13.3
% Basic CST score - English	29.5	27.6	-9.7	29.5	27.4	-10.4
Mean CST score - Math	367.8	382.1	5.3	367.8	381.0	4.9
% Advanced CST score - Math	27.2	33.7	25.5	27.2	33.8	25.7
% Basic CST score - Math	22.4	20.4	-13.3	22.4	20.8	-10.4

Data from 2013-14 from the California Department of Education. “All schools” includes 50 intervention schools and 34 comparison schools. “Matched schools” includes 34 intervention schools and 34 comparison schools. The standardized difference = $(\mu_I - \mu_C)/[(S^2_I + S^2_C)/2] \times 100$, where μ_I is the mean in the intervention group, μ_C is the mean in the comparison district, S^2_I is the standard deviation in the intervention district, and S^2_C is the standard deviation in the control group. When the SD=0, the means are equal, and when SD=100, there is a one standard deviation difference.
