# Supplementary material for: Evaluation of a city-wide school-located influenza vaccination program in Oakland, California, with respect to vaccination coverage, school absences, and laboratory-confirmed influenza: A matched cohort study
Source: PLoS Med. 2020 Aug 18;17(8):e1003238. doi: 10.1371/journal.pmed.1003238 (PMC7433855; doi:10.1371/journal.pmed.1003238)
Supplement: S2 Appendix — (PDF) [file pmed.1003238.s003.pdf]

Appendix to *Evaluation of a city-wide school-located influenza vaccination program in Oakland, California with respect to vaccination coverage, school absences, and laboratory-confirmed influenza: a matched cohort study*

S2 Appendix. Statistical power calculations

Vaccine coverage survey

To estimate the required sample size for the survey, we assumed a realistic response rate of 50 surveys per school (approximately 16% of students), type I error = 0.05, intraclass correlation coefficient = 0.01, and 70% vaccine coverage in the comparison district. Enrolling 22 school pairs (44 schools total) allowed us to detect a minimum difference in vaccination coverage between districts of 6.5% with 80% statistical power.

Influenza hospitalization

For direct effects of the program, we assessed statistical power for influenza hospitalization among children 5-14 years. Because influenza hospitalization is rare among this age group, we found that there is very little power to detect relative risks of meaningful size among elementary school-aged children (i.e., the direct effect of the program): we would only be able to detect a 95% relative reduction (RR =0.05) or more with 80% statistical power (Figure 1). For indirect effects among other age groups, statistical power is limited to detect effects smaller than RR<0.5 (Figure 2). For individuals aged 65+ years, we could detect an RR<0.6 with good statistical power, and for all individuals not of elementary school age (0-4, ≥15 years), we could detect an RR<0.75. Based on this power analysis, we plan to only estimate indirect effects of the Shoo the Flu program on influenza hospitalization among individuals 65+ years and among individuals not of elementary school age since RRs>0.6 can be detected with statistical power=0.8 for those subgroups. There was very limited statistical power to detect relative risks among intensive care unit patients with influenza-like illness (Figure 3).

Figure A. Power analysis for intervention effects on influenza hospitalization among children 5-12 years

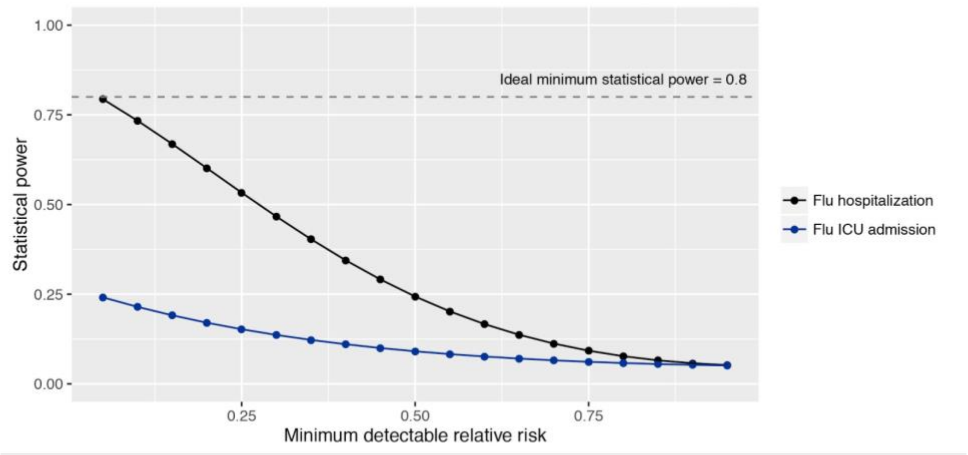

Figure B. Power analysis for indirect effects on influenza hospitalization

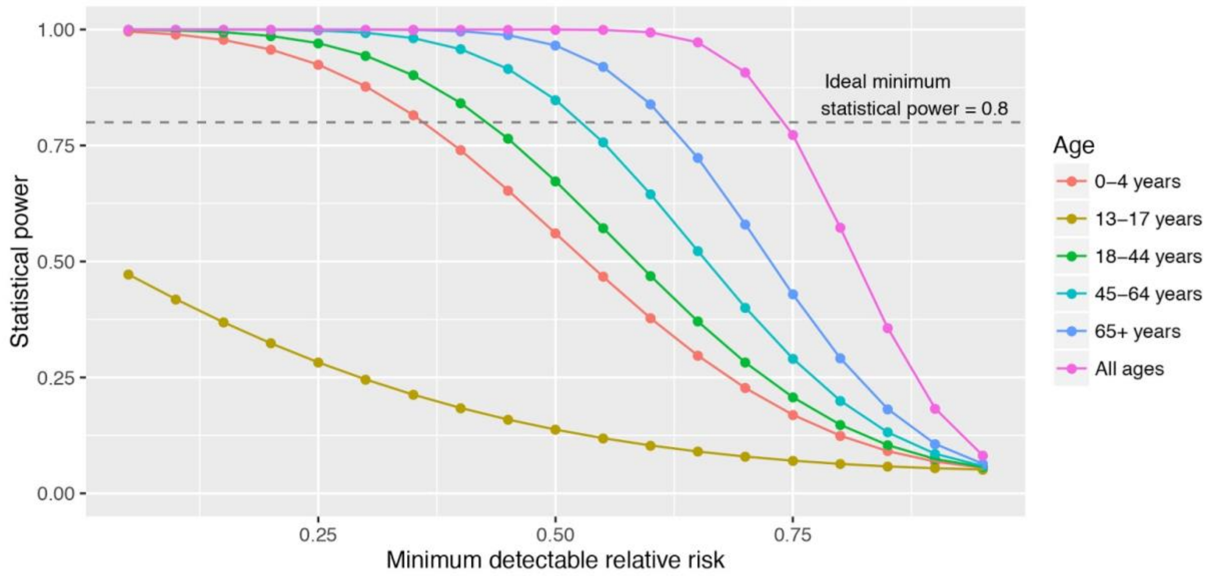

Figure C. Power analysis for indirect effects on influenza ICU admission

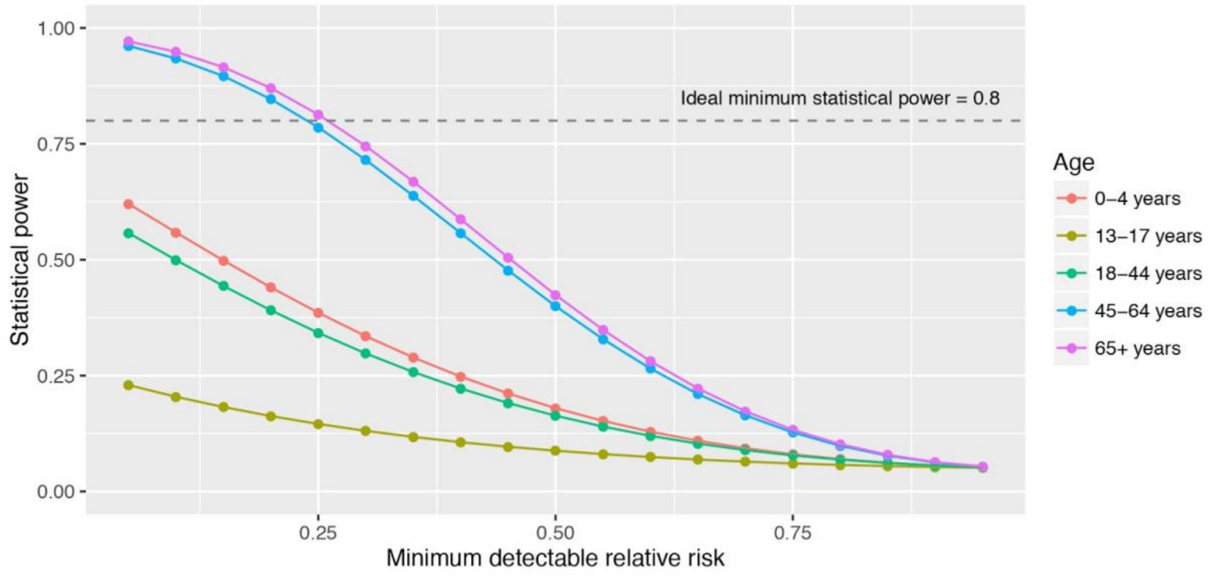

<u>School absenteeism</u>

We estimated the level of statistical power given our fixed sample size (all pre-program reported absences in in 50 OUSD schools and 34 WCCUSD schools) for different potential minimum detectable effects (Armitage et al., 2002; Chow et al., 2008; Dixon et al., 1983). We found that there is sufficient statistical power to detect mean differences in the all-cause absentee rate of approximately 0.0065 to 0.01 and differences of 0.006 to 0.01 in the illness-specific absentee rate (Figures 4-5). If we were to collect absentee data from additional control

schools in other districts, there would be relatively small gains in statistical power. Thus, we plan to proceed with the analysis in OUSD and WCCUSD.

Figure D. Power analysis for illness-specific absence rate

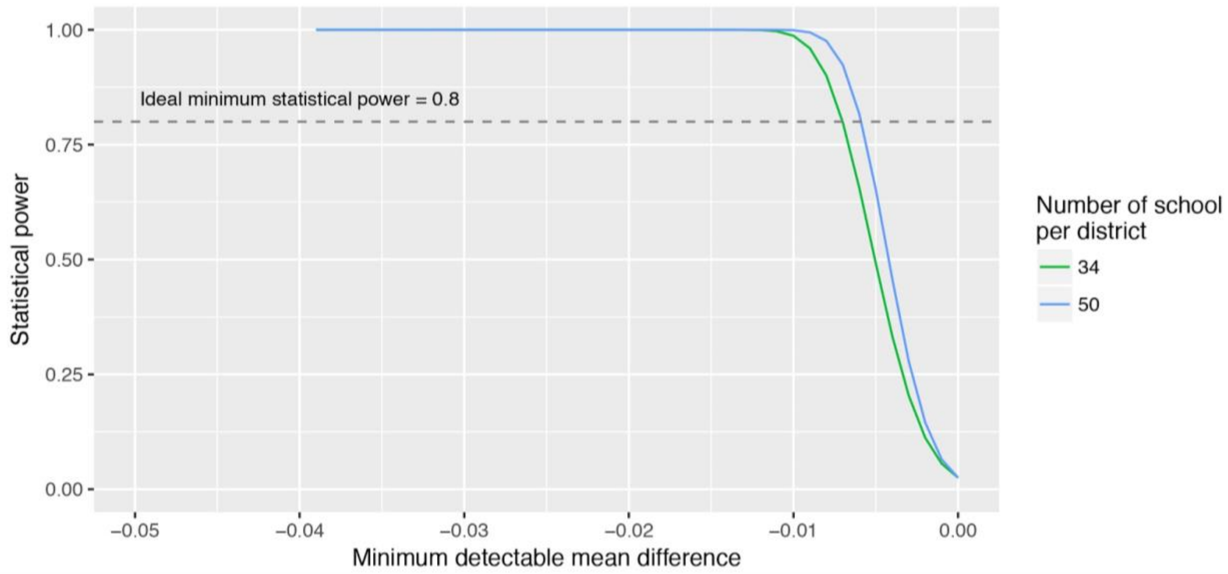

Figure E. Power analysis for all-cause absence rate

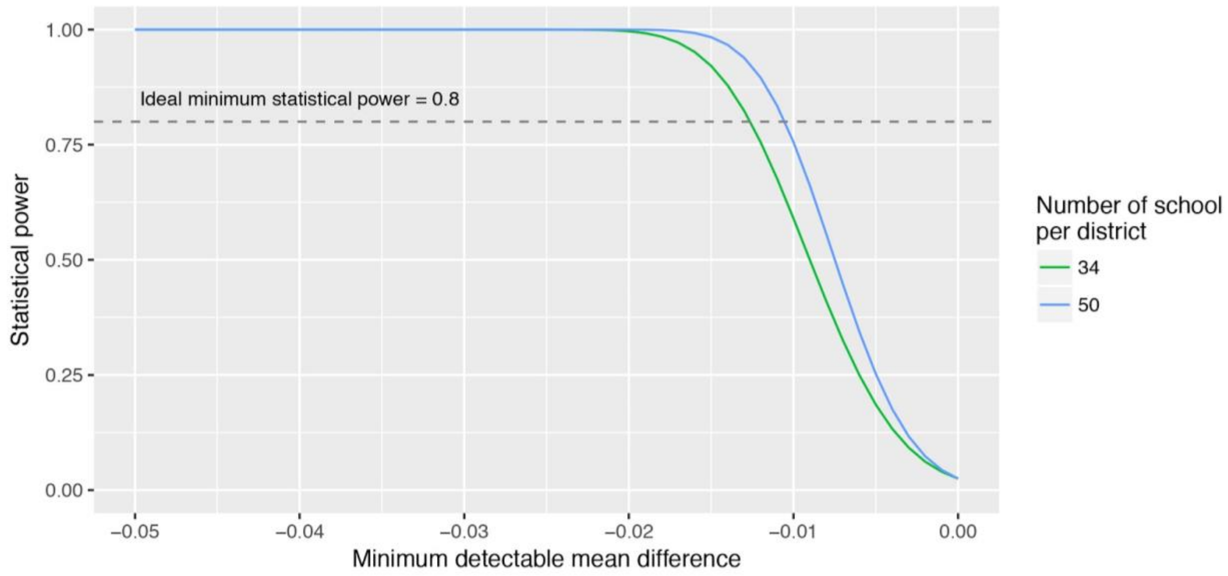

Armitage, P., G. Berry, and J. N. S. Matthews. Statistical Methods in Medical Research. Oxford: Blackwell; 2002.

Chow, S.-C., J. Shao, and H. Wang. Sample Size Calculations in Clinical Research. New York: Marcel Dekker; 2008.

Dixon, W. J., and F. J. Massey, Jr. Introduction to Statistical Analysis. New York: McGraw-Hill; 1983.
