# Supplementary material for: Evaluation of a city-wide school-located influenza vaccination program in Oakland, California, with respect to vaccination coverage, school absences, and laboratory-confirmed influenza: A matched cohort study
Source: PLoS Med. 2020 Aug 18;17(8):e1003238. doi: 10.1371/journal.pmed.1003238 (PMC7433855; doi:10.1371/journal.pmed.1003238)
Supplement: S3 Appendix — (PDF) [file pmed.1003238.s004.pdf]

Appendix to *Evaluation of a city-wide school-located influenza vaccination program in Oakland, California with respect to vaccination coverage, school absences, and laboratory-confirmed influenza: a matched cohort study*

S3 Appendix. Assessment of selection bias in the vaccine coverage survey

It is possible that selection bias may have occurred if the parents who chose to respond to our surveys were not representative of the overall parent population in each of the participating schools or in the districts as a whole. We assessed possible selection bias by comparing the percentage of parents in different race and education categories in our survey sample to the pre-program percentages for the participating 44 schools and for the entire districts using data from the California Department of Education. We found the distributions of student race and parent education in our sample differed from those in participating schools and the districts as a whole, as shown in the figures below. These figures only show demographics from the 2017 survey, but 2018 survey results are very similar.

Fig A. Distribution of parent's highest education level in 2017 survey sample and in all 44 schools in the sample and in the entire districts based on pre-intervention data from the California Department of Education

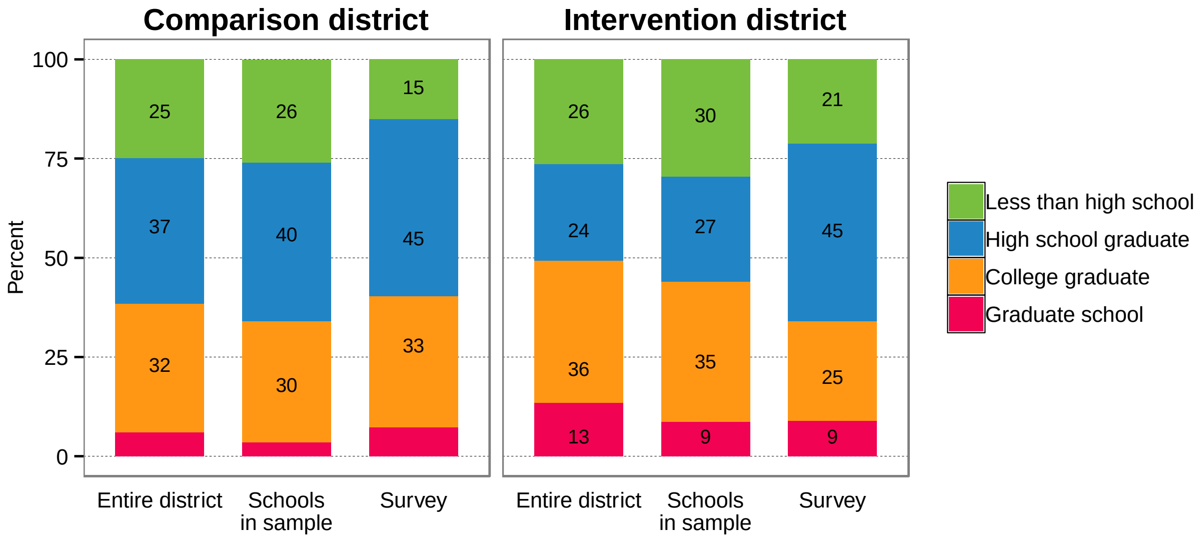

"Entire district" includes pre-intervention caregiver education in all elementary schools in each district; "Schools in sample" includes pre-intervention caregiver education in schools in which surveys were administered; "Survey" includes caregiver education in responses to our survey.

Fig B. Distribution of student race in our survey sample and in all 44 schools in the sample and in the entire districts based on pre-intervention data from the California Department of Education

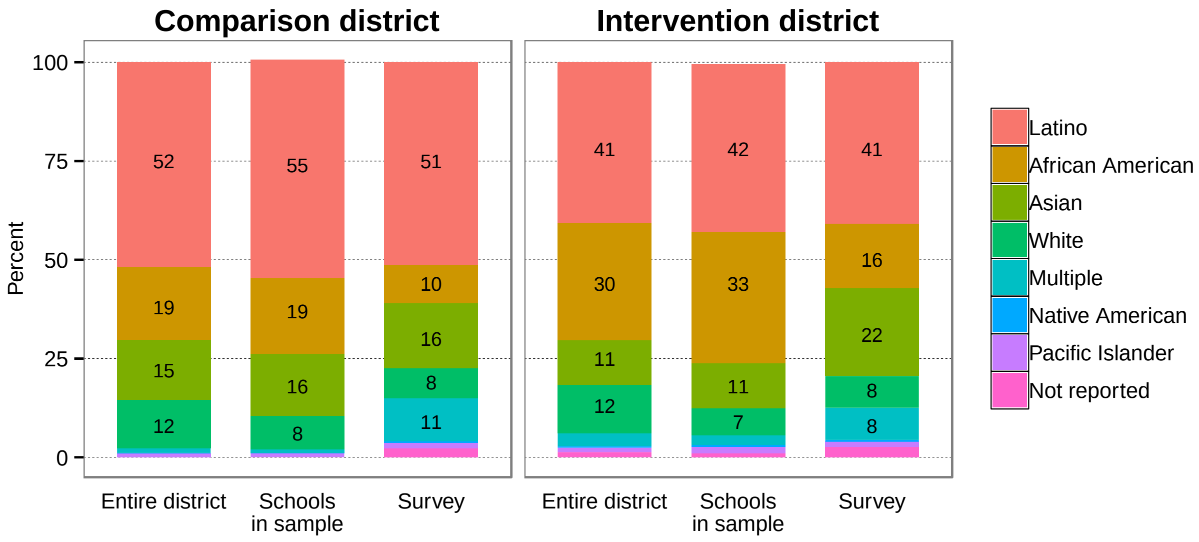

"Entire district" includes pre-intervention student race in all elementary schools in each district; "Schools in sample" includes pre-intervention student race in schools in which surveys were administered; "Survey" includes student race in responses to our survey

Information on the joint distribution of race and education is not available from the department of education, so we re-weighted results by race and education separately using two references: 1) All schools in each district, 2) 44 schools in the sample. This step applied the percentage vaccinated within each race or education group to the percentage in each race or education group in the two reference populations.
