# Supplementary material for: Evaluation of a city-wide school-located influenza vaccination program in Oakland, California, with respect to vaccination coverage, school absences, and laboratory-confirmed influenza: A matched cohort study
Source: PLoS Med. 2020 Aug 18;17(8):e1003238. doi: 10.1371/journal.pmed.1003238 (PMC7433855; doi:10.1371/journal.pmed.1003238)
Supplement: S4 Appendix — (PDF) [file pmed.1003238.s005.pdf]

Appendix to *Evaluation of a city-wide school-located influenza vaccination program in Oakland, California with respect to vaccination coverage, school absences, and laboratory-confirmed influenza: a matched cohort study*

S4 Appendix. Pre-intervention influenza hospitalization and school absence rates in each site

Figure A. Weekly incidence of inpatient laboratory-confirmed influenza prior to and during the intervention among non-elementary aged individuals

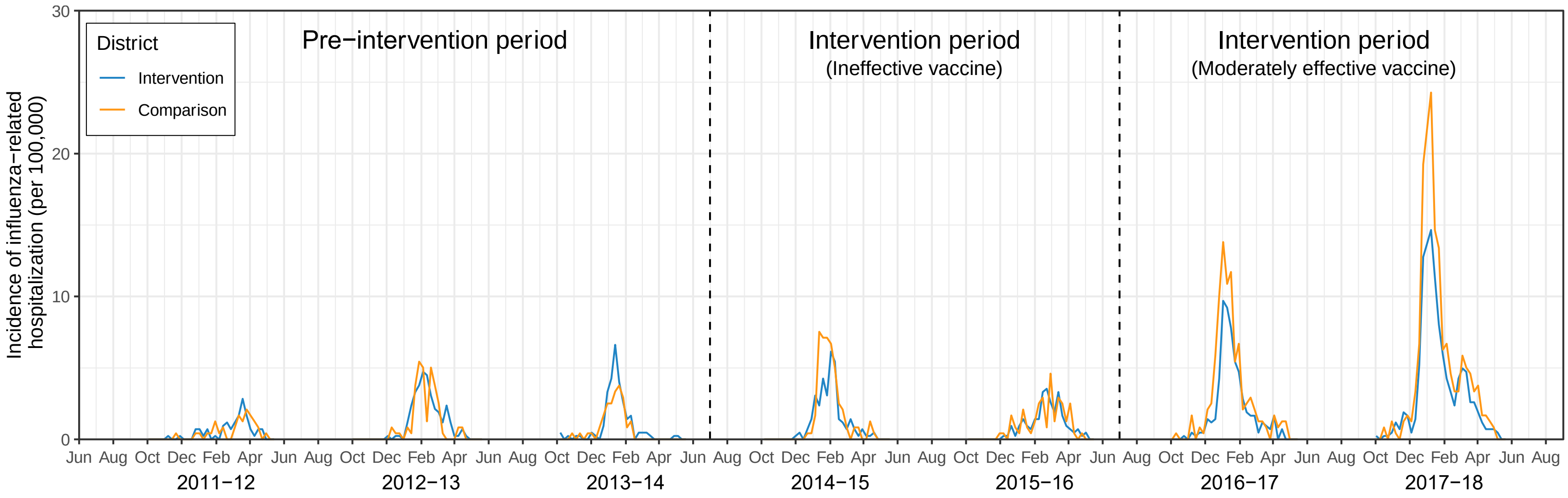

Weekly incidence proportion of laboratory-confirmed influenza hospitalizations, including intensive care unit admissions, between Week 40-52 and Week 1-20 of each year. Hospitalizations included school district residents tested at health care facility laboratories located in zip codes overlapping with OUSD and WCCUSD (Alameda County Public Health Department, Children's Hospital Oakland, Contra Costa Public Health Department, Kaiser Permanente, Sutter Health). Population denominators were obtained from the U.S. 2010 Census using the same set of zip codes.

Figure B. Weekly incidence of inpatient laboratory-confirmed influenza prior to and during the intervention among elderly individuals

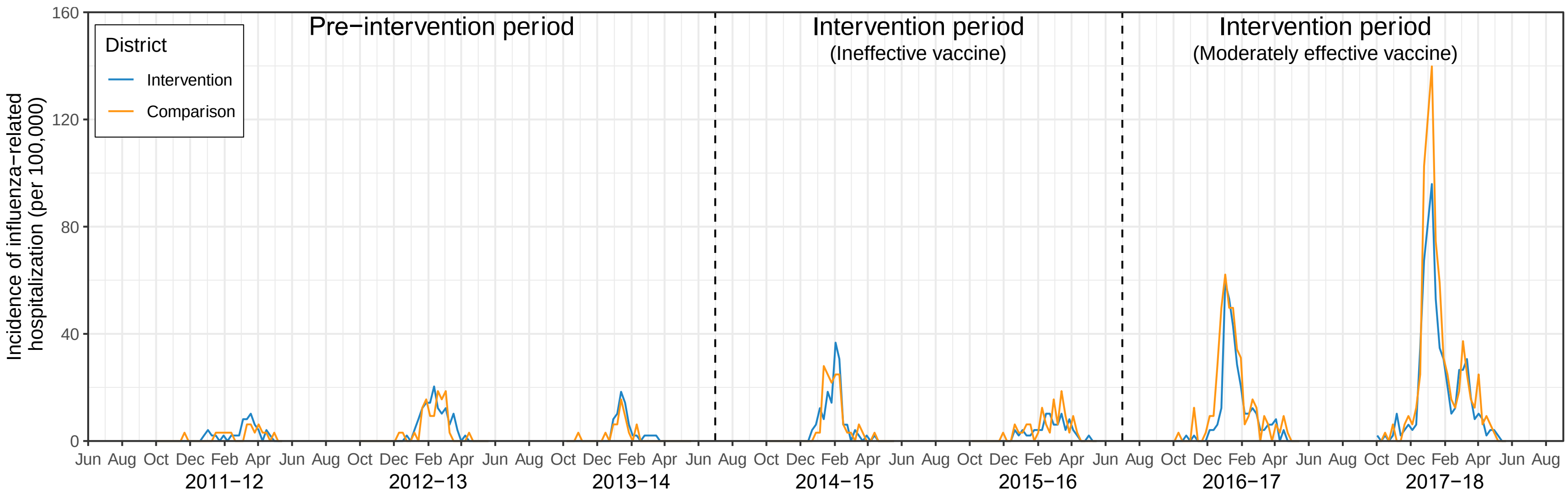

Weekly incidence proportion of laboratory-confirmed influenza hospitalizations, including intensive care unit admissions, between Week 40-52 and Week 1-20 of each year. Hospitalizations included school district residents tested at health care facility laboratories located in zip codes overlapping with OUSD and WCCUSD (Alameda County Public Health Department, Children's Hospital Oakland, Contra Costa Public Health Department, Kaiser Permanente, Sutter Health). Population denominators were obtained from the U.S. 2010 Census using the same set of zip codes.

Figure C. Mean pre-intervention daily all-cause absences by school district

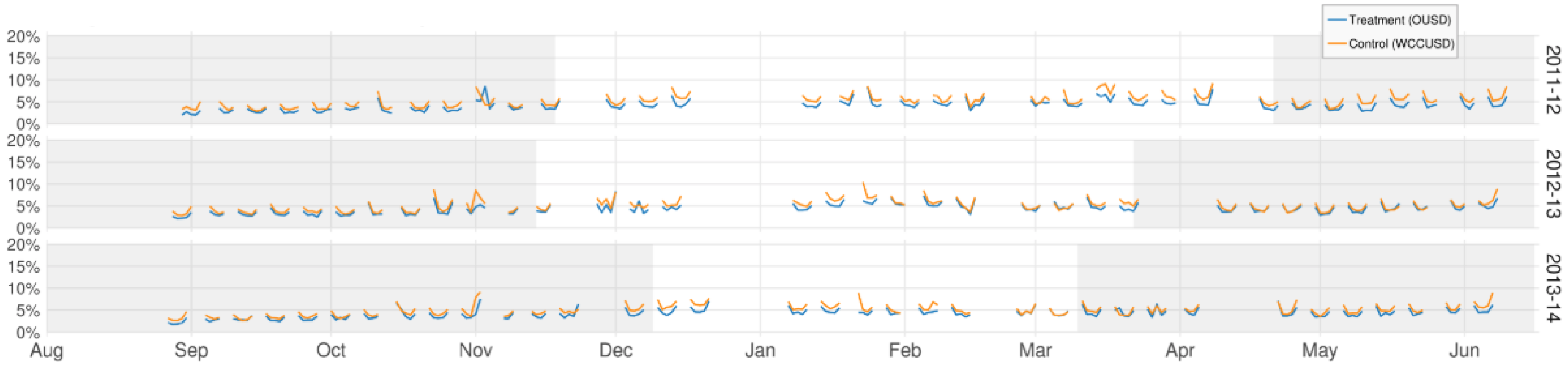

Gray areas indicate weeks outside of influenza season, and white areas indicate weeks inside of influenza season according to our definition based on the frequency of influenza-like illness visits in California.

Figure D. Mean pre-intervention daily illness-specific absences by school district

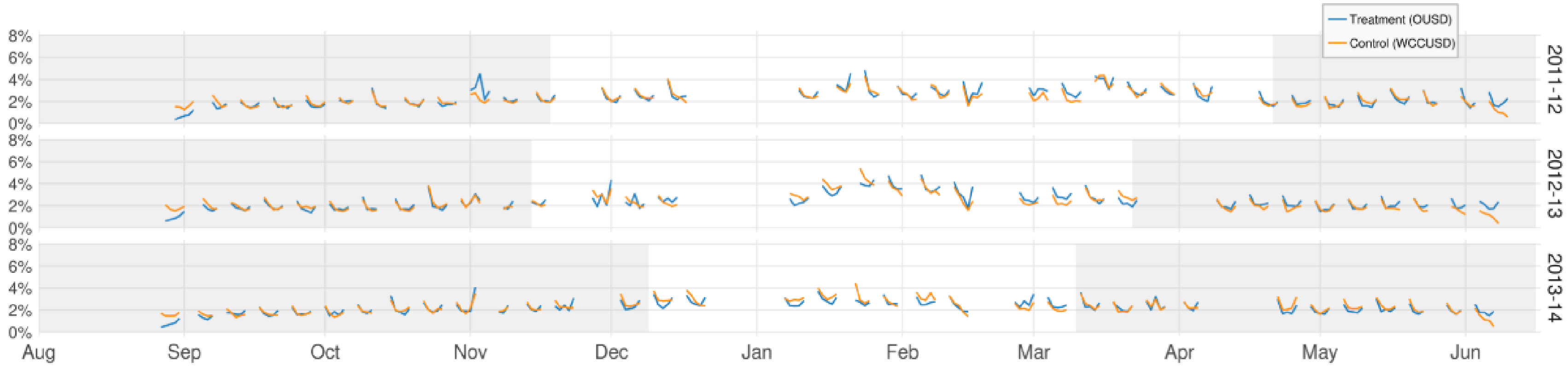

Gray areas indicate weeks outside of influenza season, and white areas indicate weeks inside of influenza season according to our definition based on the frequency of influenza-like illness visits in California.
