# Supplementary material for: Evaluation of a city-wide school-located influenza vaccination program in Oakland, California, with respect to vaccination coverage, school absences, and laboratory-confirmed influenza: A matched cohort study
Source: PLoS Med. 2020 Aug 18;17(8):e1003238. doi: 10.1371/journal.pmed.1003238 (PMC7433855; doi:10.1371/journal.pmed.1003238)
Supplement: S5 Appendix — (PDF) [file pmed.1003238.s006.pdf]

Appendix to *Evaluation of a city-wide school-located influenza vaccination program in Oakland, California with respect to vaccination coverage, school absences, and laboratory-confirmed influenza: a matched cohort study*

S5 Appendix. Quantitative bias analysis to assess misclassification of absence rates

Through conversations with each school district, we assumed the sensitivity and the false positive rate of absence classification followed beta distributions with the parameters in the table below. The false positive rate was defined as the number of student-days misclassified as absent when the child was present divided by the total number of student-days.

Table A. Assumed distributions of sensitivity and specificity in the probabilistic bias analysis

	**All-cause absences**	**Illness-specific absences**
**Sensitivity of outcome classification**		
Intervention	$\alpha = 14, \beta = 1$ (2011-2016)<br>$\alpha = 6, \beta = 1$ (2017-18)*	$\alpha = 3, \beta = 1.5$
Comparison	$\alpha = 6, \beta = 1$	$\alpha = 3, \beta = 1.5$
**False positive rate**		
Intervention	$\alpha = 2, \beta = 19$<br>Scaled by 300	$\alpha = 2, \beta = 5$<br>Scaled by 300
Comparison	$\alpha = 2, \beta = 5$<br>Scaled by 300	$\alpha = 1.5, \beta = 2$<br>Scaled by 400

* Sensitivity distributions differ for the intervention district by year because the district informed us that classification had improved in 2017-18.
