# Supplementary material for: Evaluation of a city-wide school-located influenza vaccination program in Oakland, California, with respect to vaccination coverage, school absences, and laboratory-confirmed influenza: A matched cohort study
Source: PLoS Med. 2020 Aug 18;17(8):e1003238. doi: 10.1371/journal.pmed.1003238 (PMC7433855; doi:10.1371/journal.pmed.1003238)
Supplement: S1 Fig — (PDF) [file pmed.1003238.s007.pdf]

Appendix to *Evaluation of a city-wide school-located influenza vaccination program in Oakland, California with respect to vaccination coverage, school absences, and laboratory-confirmed influenza: a matched cohort study*

**S1 Figure. Alternative influenza season definitions**

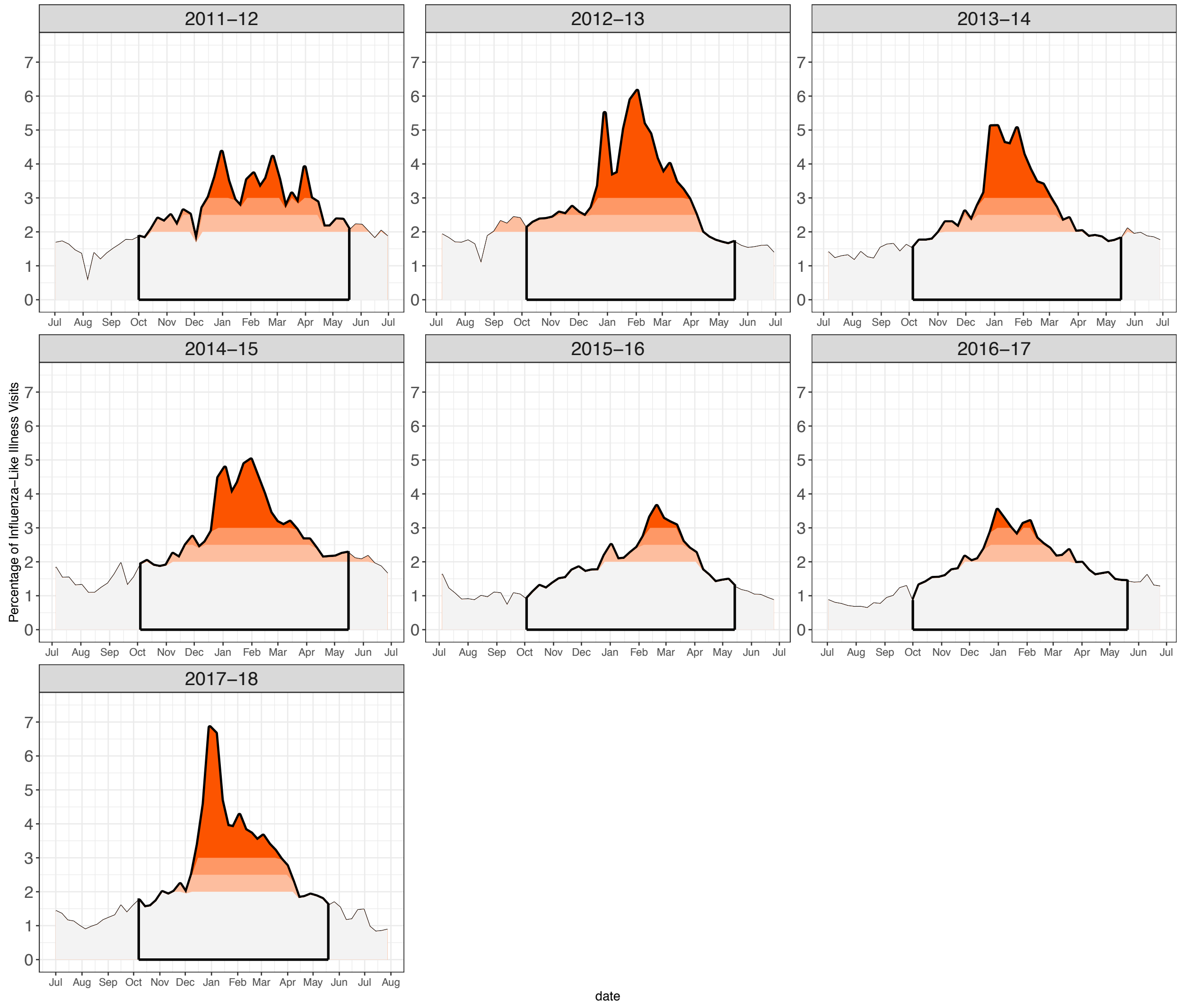


Each panel plots the percentage of influenza-like illness associated visits to health care providers in California (source: California Department of Public Health). The red colors indicate the influenza season definitions used in this analysis. Influenza season started when there were at least two consecutive weeks in which the percentage of medical visits for influenza-like illness in California as reported as reported by the California Department of Public Health exceeded a cutoff, and the season ended when there were at least two consecutive weeks in which the percentage was less than or equal to a cutoff. We examined seasons defined using cutoffs of 2% (lightest shade of red), 2.5% (medium shade of red), and 3% (darkest shade of red). The weeks inside the period of time outlined in the thick black line are classified as influenza season by the US Centers for Disease Control.
