# Supplementary material for: Evaluation of a city-wide school-located influenza vaccination program in Oakland, California, with respect to vaccination coverage, school absences, and laboratory-confirmed influenza: A matched cohort study
Source: PLoS Med. 2020 Aug 18;17(8):e1003238. doi: 10.1371/journal.pmed.1003238 (PMC7433855; doi:10.1371/journal.pmed.1003238)
Supplement: S3 Fig — (PDF) [file pmed.1003238.s009.pdf]

Appendix to *Evaluation of a city-wide school-located influenza vaccination program in Oakland, California with respect to vaccination coverage, school absences, and laboratory-confirmed influenza: a matched cohort study*

S3 Figure. Standardized percent of students vaccinated for influenza from all sources among elementary school students in 44 OUSD and WCCUSD schools for 2014-2018

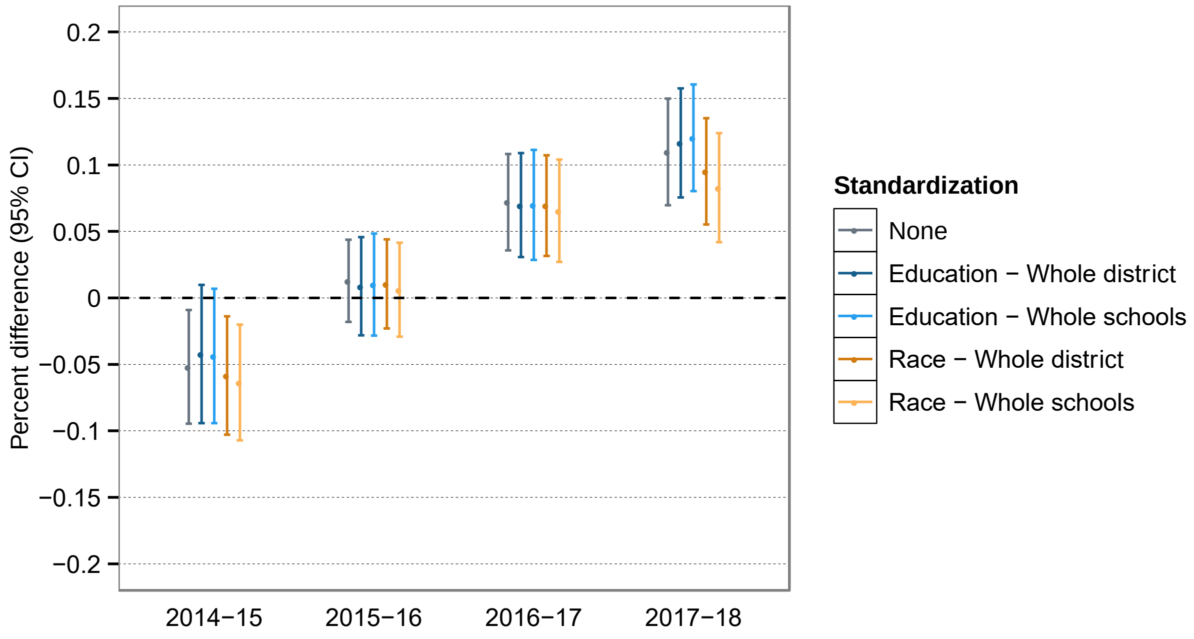

“Whole district” indicates that the data was re-weighted to represent the caregiver education level or student race distributions in the entire school district (50 intervention schools, 34 comparison schools). “Whole schools” indicates that the data was re-weighted to represent the caregiver education level or student race distributions in all the 44 matched schools included in the vaccine coverage survey.
