# Supplementary material for: Evaluation of a city-wide school-located influenza vaccination program in Oakland, California, with respect to vaccination coverage, school absences, and laboratory-confirmed influenza: A matched cohort study
Source: PLoS Med. 2020 Aug 18;17(8):e1003238. doi: 10.1371/journal.pmed.1003238 (PMC7433855; doi:10.1371/journal.pmed.1003238)
Supplement: S6 Fig — (PDF) [file pmed.1003238.s012.pdf]

Appendix to *Evaluation of a city-wide school-located influenza vaccination program in Oakland, California with respect to vaccination coverage, school absences, and laboratory-confirmed influenza: a matched cohort study*

S6 Figure. Relationship between caregiver-reported influenza vaccination coverage among elementary students and school-level participation in the SLIV program in intervention schools

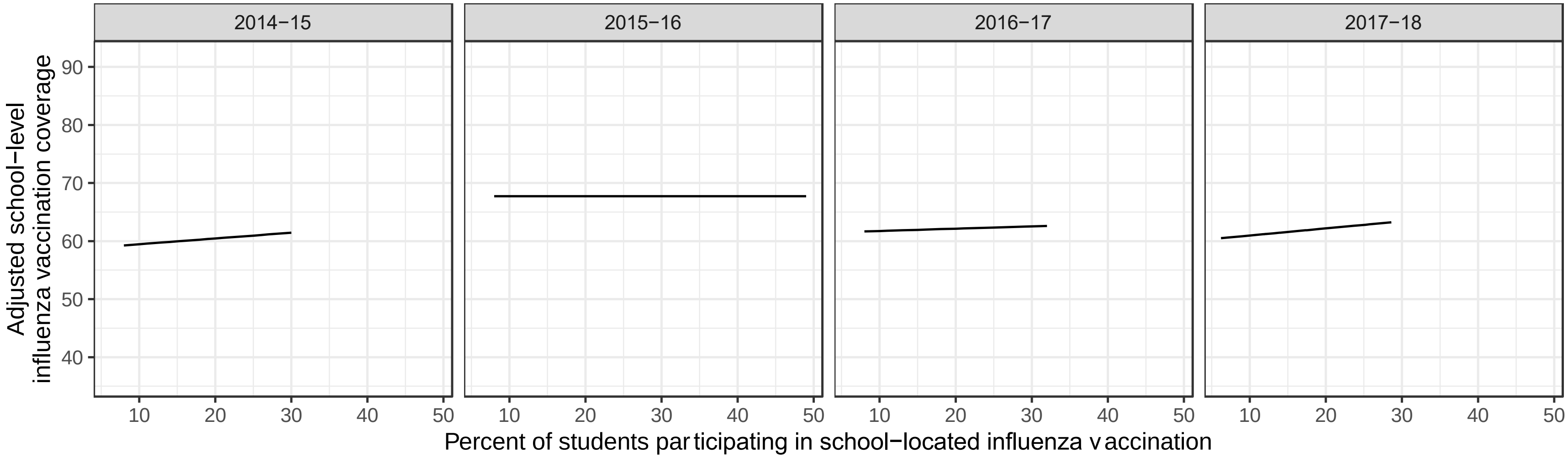

Each point is the school-level influenza vaccination coverage by elementary students by the percentage of students participating in school-located influenza vaccination program in the intervention district. The line is the predicted fit from an ensemble machine learning model that adjusted for student race and grade and mean school-level characteristics including enrollment, class size, parental education, academic performance index scores, California standardized test scores, and the school-level percentage of English language learners and percentage of students receiving free lunch at school.
