# Supplementary material for: Evaluation of a city-wide school-located influenza vaccination program in Oakland, California, with respect to vaccination coverage, school absences, and laboratory-confirmed influenza: A matched cohort study
Source: PLoS Med. 2020 Aug 18;17(8):e1003238. doi: 10.1371/journal.pmed.1003238 (PMC7433855; doi:10.1371/journal.pmed.1003238)
Supplement: S7 Fig — (PDF) [file pmed.1003238.s013.pdf]

Appendix to *Evaluation of a city-wide school-located influenza vaccination program in Oakland, California with respect to vaccination coverage, school absences, and laboratory-confirmed influenza: a matched cohort study*

S7 Figure. Overall and indirect effects on cumulative incidence of inpatient laboratory-confirmed influenza during the peak week of influenza hospitalization

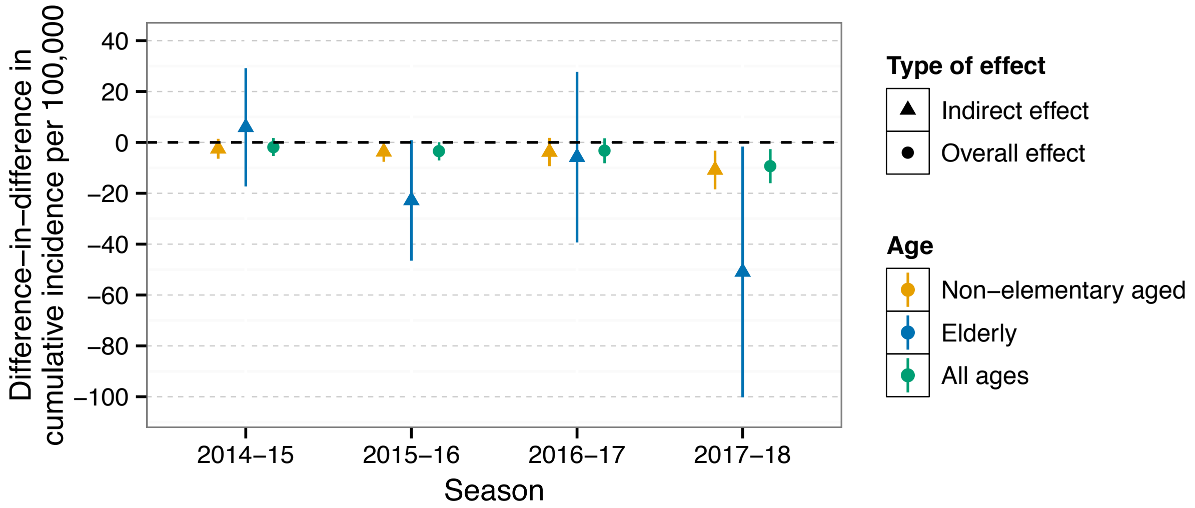

Cumulative incidence of laboratory-confirmed influenza hospitalization and intensive care unit admission during the week with the highest influenza hospitalization. Difference-in-difference estimates represent the difference between intervention and control groups in their change in incidence from the three pre-program years (2011-2013) to each program year, which removes any time-invariant differences between groups (measured or unmeasured). Parameters were estimated using a log-linear Poisson model with an offset for population size, and were further adjusted for age, race, and sex. Standard errors and 95% confidence intervals were obtained using the delta method.
