# Supplementary material for: Evaluation of a city-wide school-located influenza vaccination program in Oakland, California, with respect to vaccination coverage, school absences, and laboratory-confirmed influenza: A matched cohort study
Source: PLoS Med. 2020 Aug 18;17(8):e1003238. doi: 10.1371/journal.pmed.1003238 (PMC7433855; doi:10.1371/journal.pmed.1003238)
Supplement: S9 Fig — (PDF) [file pmed.1003238.s015.pdf]

Appendix to *Evaluation of a city-wide school-located influenza vaccination program in Oakland, California with respect to vaccination coverage, school absences, and laboratory-confirmed influenza: a matched cohort study*

S9 Figure. Overall and indirect effects on length of influenza hospitalization including outlier

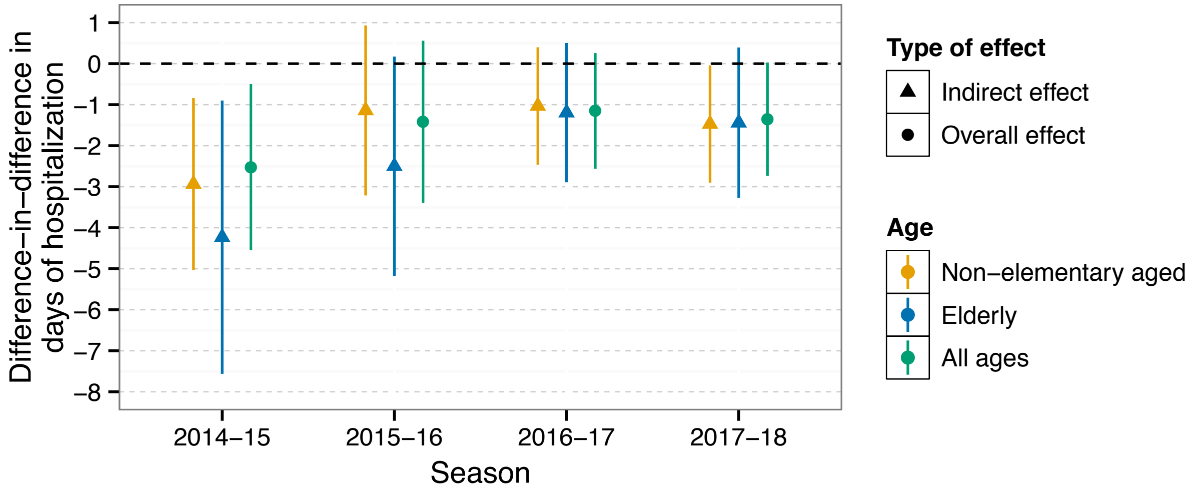

Weekly incidence proportion of laboratory-confirmed influenza hospitalization and intensive care unit admission. Analyses were restricted to influenza season, defined as the period following at least two consecutive weeks in which the percentage of medical visits for influenza-like illness in California as reported by the California Department of Public Health exceeded 2.5% and prior to at least two consecutive weeks in which the percentage was less than or equal to 2.5%. Influenza case counts were obtained from patients at health care facility laboratories performing influenza testing in zip codes overlapping with OUSD and WCCUSD (Alameda County Public Health Department, Children's Hospital Oakland, Contra Costa Public Health Department, Kaiser Permanente, Sutter Health). Population denominators were obtained from the U.S. 2010 Census using the same set of zip codes. Includes one observation among an individual over age 65 years in the comparison site in 2014-15 with a length of stay of 117 days.
