# Supplementary material for: Evaluation of a city-wide school-located influenza vaccination program in Oakland, California, with respect to vaccination coverage, school absences, and laboratory-confirmed influenza: A matched cohort study
Source: PLoS Med. 2020 Aug 18;17(8):e1003238. doi: 10.1371/journal.pmed.1003238 (PMC7433855; doi:10.1371/journal.pmed.1003238)
Supplement: S12 Fig — (PDF) [file pmed.1003238.s018.pdf]

Appendix to *Evaluation of a city-wide school-located influenza vaccination program in Oakland, California with respect to vaccination coverage, school absences, and laboratory-confirmed influenza: a matched cohort study*

S12 Figure. Sensitivity analyses estimating overall and indirect effects on cumulative incidence of inpatient laboratory-confirmed influenza with alternative numerators and denominators

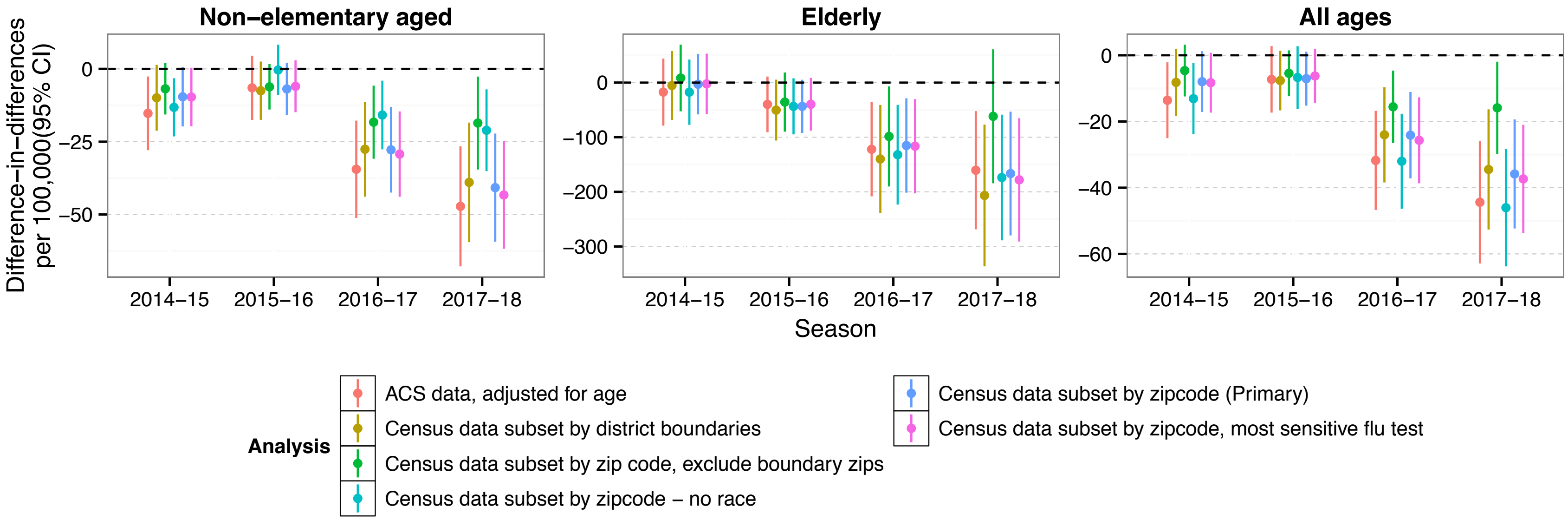

Cumulative incidence of laboratory-confirmed influenza hospitalization and intensive care unit admission. Analyses were restricted to influenza season, defined as the period following at least two consecutive weeks in which the percentage of medical visits for influenza-like illness in California as reported by the California Department of Public Health exceeded 2.5% and prior to at least two consecutive weeks in which the percentage was less than or equal to 2.5%. Differences-in-differences were adjusted for age, race, and sex and compare the difference in incidence in the intervention group during a program year to the incidence in the intervention group in three pre-program years (2011-2013) to the analogous differences in the comparison group. Parameters were estimated using a log-linear Poisson model with an offset for population size. Standard errors and 95% confidence intervals were obtained using the delta method.

Census data subset by zip code (Primary analysis): Influenza case counts were obtained from patients at health care facility laboratories performing influenza testing in zip codes overlapping with OUSD and WCCUSD (Alameda County Public Health Department, Children's Hospital Oakland, Contra Costa Public Health Department, Kaiser Permanente, Sutter Health). Population denominators were obtained from the U.S. 2010 Census using the same set of zip codes.

Census data subset by zip code, no race: The primary analysis adjusted for race, and patients with missing observations were dropped from the analysis. This analysis includes those patients but does not adjust for race.

ACS data, adjusted for age: Population denominators were obtained from the American Community Survey 5-year estimates and subset by school district boundaries.

Census data subset by district boundaries: Population denominators were obtained from the U.S. 2010 Census and subset by school district boundaries.

Census data subset by zip code, exclude boundary zips: Population denominators were obtained from the U.S. 2010 Census using the same set of zip codes. Zip codes that overlap with school district boundaries were excluded from both case counts and population denominators.
