# Supplementary material for: Evaluation of a city-wide school-located influenza vaccination program in Oakland, California, with respect to vaccination coverage, school absences, and laboratory-confirmed influenza: A matched cohort study
Source: PLoS Med. 2020 Aug 18;17(8):e1003238. doi: 10.1371/journal.pmed.1003238 (PMC7433855; doi:10.1371/journal.pmed.1003238)
Supplement: S13 Fig — (PDF) [file pmed.1003238.s019.pdf]

Appendix to *Evaluation of a city-wide school-located influenza vaccination program in Oakland, California with respect to vaccination coverage, school absences, and laboratory-confirmed influenza: a matched cohort study*

S13 Figure. Sensitivity analyses estimating overall and indirect effects on cumulative incidence of inpatient laboratory-confirmed influenza using alternative influenza season definitions

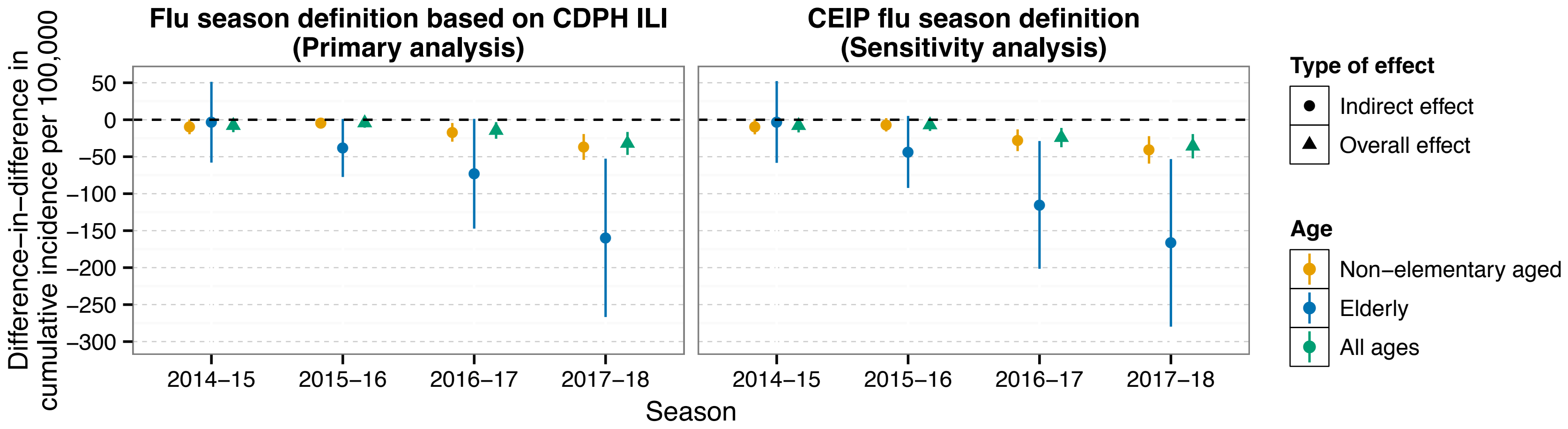

Cumulative incidence of laboratory-confirmed influenza hospitalization and intensive care unit admission. Differences-in-differences were adjusted for age, race, and sex and compare the difference in incidence in the intervention group during a program year to the incidence in the intervention group in three pre-program years (2011-2013) to the analogous differences in the comparison group. Parameters were estimated using a log-linear Poisson model with an offset for population size. Standard errors and 95% confidence intervals were obtained using the delta method.

Flu season definition based on CDPH ILI (Primary analysis): This definition is based on the percentage of medical visits for influenza-like illness in California as reported by the California Department of Public Health. Influenza season started when there were at least 2 consecutive weeks in which the percentage of medical visits for influenza-like illness exceeded 2.5%, and the season ended when there were at least two consecutive weeks in which the percentage was less than or equal to 2.5%

CEIP flu season definition (Sensitivity analysis): The period from October 1 to April 30 was classified as influenza season.
