# Supplementary material for: Evaluation of a city-wide school-located influenza vaccination program in Oakland, California, with respect to vaccination coverage, school absences, and laboratory-confirmed influenza: A matched cohort study
Source: PLoS Med. 2020 Aug 18;17(8):e1003238. doi: 10.1371/journal.pmed.1003238 (PMC7433855; doi:10.1371/journal.pmed.1003238)
Supplement: S14 Fig — (PDF) [file pmed.1003238.s020.pdf]

Appendix to *Evaluation of a city-wide school-located influenza vaccination program in Oakland, California with respect to vaccination coverage, school absences, and laboratory-confirmed influenza: a matched cohort study*

S14 Figure. Sensitivity analyses estimating difference-in-differences in school absence rates using alternative influenza season definitions

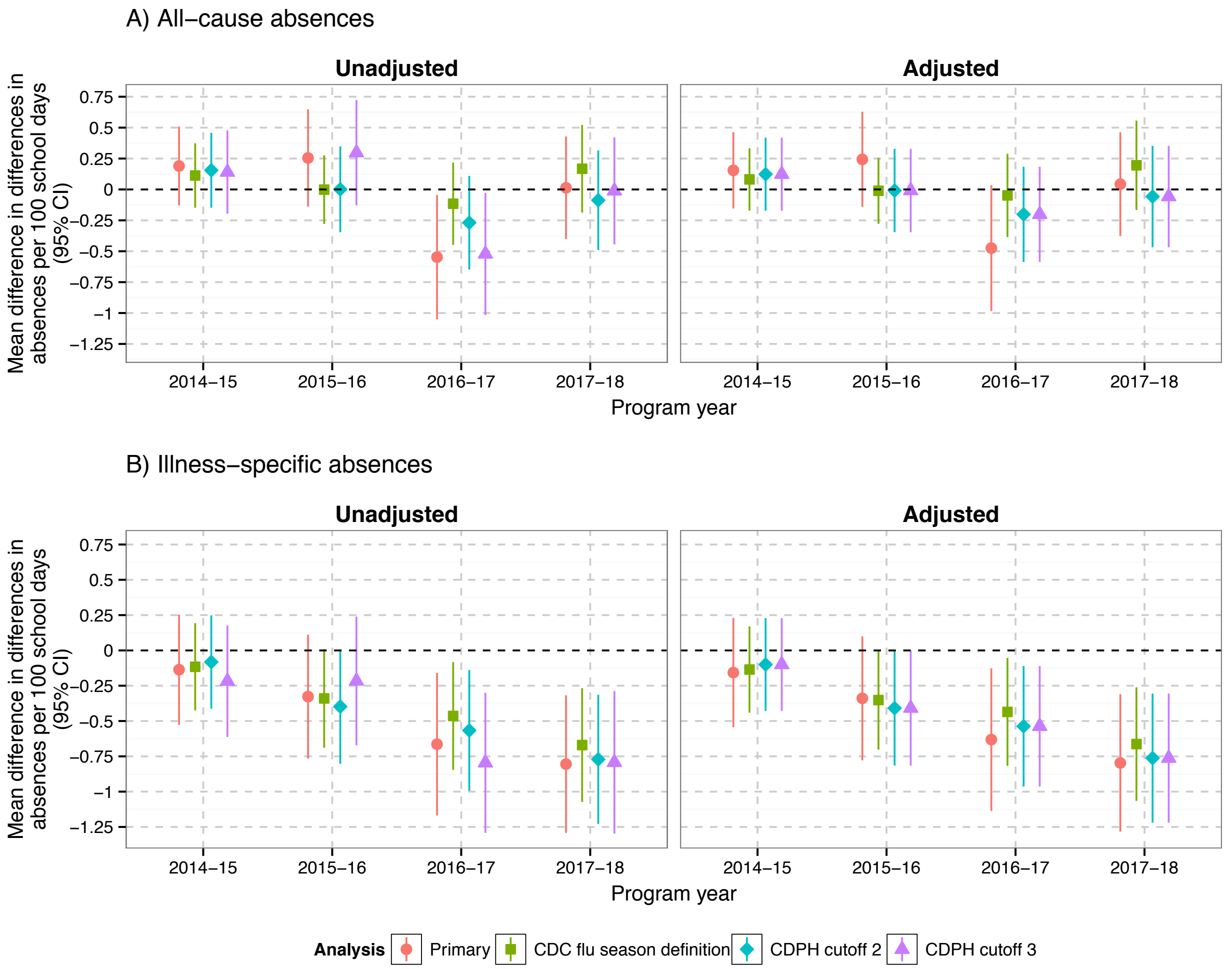

Each difference-in-difference estimate compares the difference in absence rates in each district in a program year compared to the three pre-program years (2011-2013), which removes any time-invariant differences between groups (measured or unmeasured). The "Primary" analysis restricted to influenza season, defined as the period following at least two consecutive weeks in which the percentage of medical visits for influenza-like illness in California as reported by the California Department of Public Health exceeded 2.5% and prior to at least two consecutive weeks in which the percentage was less than or equal to 2.5%. The "CDC flu season definition" included the period after week 40 and before week 20 of each year. The "CDPH cutoff" analyses restricted to influenza season using the same definition of influenza season as in the primary analysis but used cutoffs of 2% and 3% instead of 2.5%. Parameters were estimated using a generalized linear model. The left-hand panels ("Unadjusted") include results for unadjusted models that did not adjust for month, student race, and grade. The right-hand panels ("Adjusted") include results for models that adjusted for month, student race, and grade. Standard errors and 95% confidence intervals account for clustering at the school level.
