# Supplementary material for: Evaluation of a city-wide school-located influenza vaccination program in Oakland, California, with respect to vaccination coverage, school absences, and laboratory-confirmed influenza: A matched cohort study
Source: PLoS Med. 2020 Aug 18;17(8):e1003238. doi: 10.1371/journal.pmed.1003238 (PMC7433855; doi:10.1371/journal.pmed.1003238)
Supplement: S16 Fig — (PDF) [file pmed.1003238.s022.pdf]

Appendix to *Evaluation of a city-wide school-located influenza vaccination program in Oakland, California with respect to vaccination coverage, school absences, and laboratory-confirmed influenza: a matched cohort study*

S16 Figure. Intervention effects on the school absence rate per 100 school days stratifying by month and influenza season

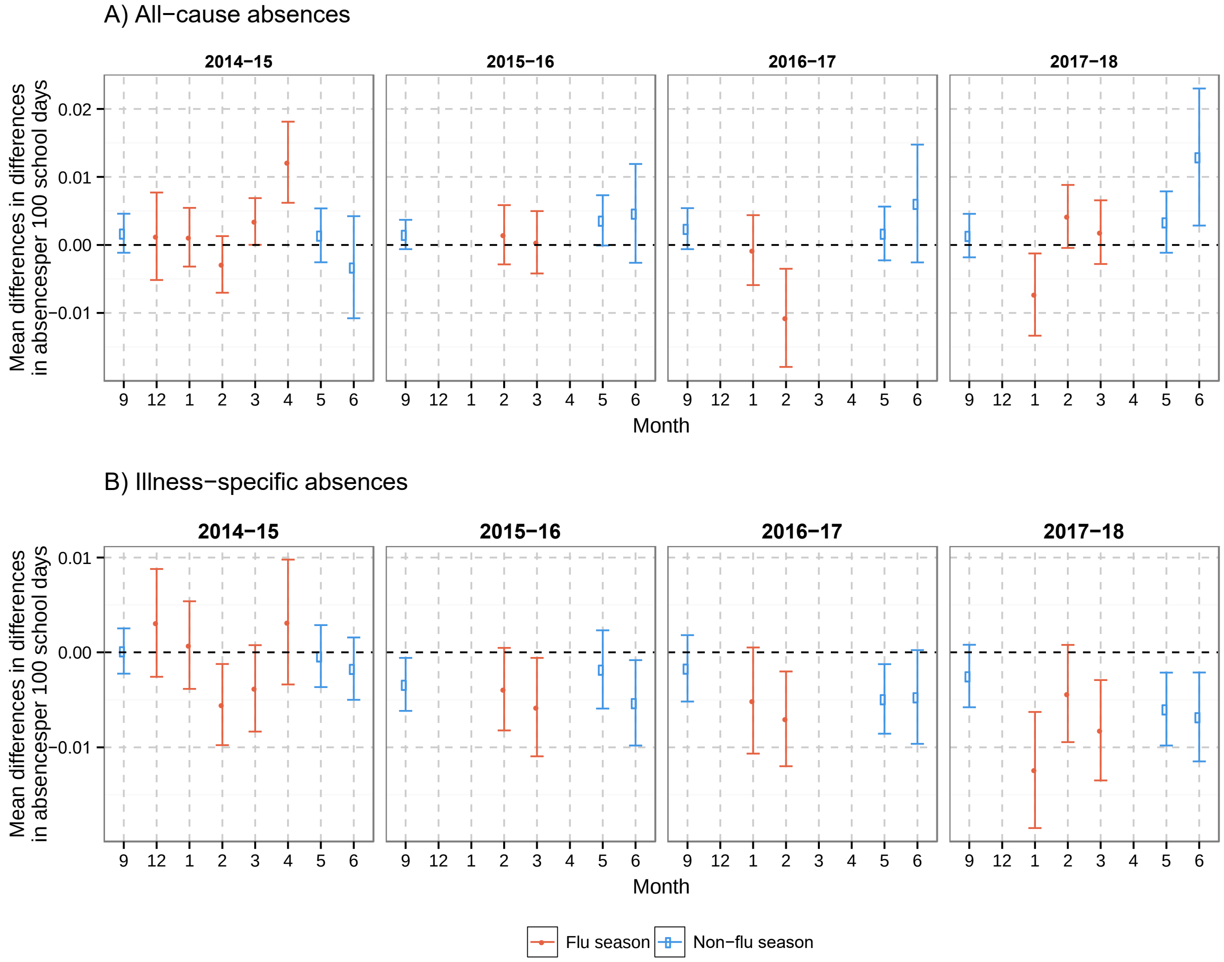

C) Causal diagram depicting hypothesized differential measurement error and negative control outcome

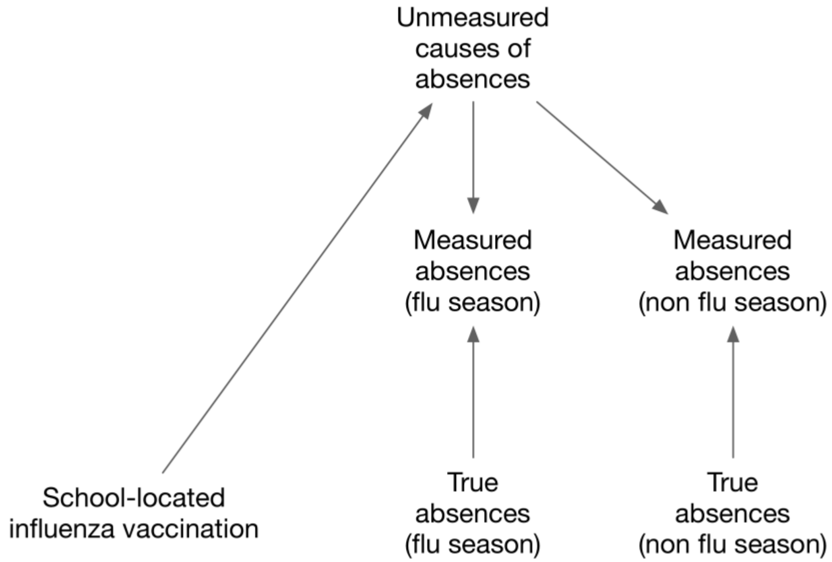

Panels A) and B) depict month-specific estimates in red are those from flu season according to the definition in the primary analysis. Month-specific estimates in blue are from the negative control time period analysis, which included September, May, and June. Each difference-in-difference estimate compares the difference in absence rates in each district in a program year compared to the three pre-program years (2011-2013), which removes any time-invariant differences between groups (measured or unmeasured). Parameters were estimated using a generalized linear model and were adjusted for student race and grade. Standard errors and 95% confidence intervals account for clustering at the school level. Panel C) presents the causal diagram we posit for the relationship between the SLIV intervention, true, and measured absences during influenza season (main outcome) and outside of influenza season (negative control outcome).
