# Supplementary material for: Evaluation of a city-wide school-located influenza vaccination program in Oakland, California, with respect to vaccination coverage, school absences, and laboratory-confirmed influenza: A matched cohort study
Source: PLoS Med. 2020 Aug 18;17(8):e1003238. doi: 10.1371/journal.pmed.1003238 (PMC7433855; doi:10.1371/journal.pmed.1003238)
Supplement: S1 Table — (PDF) [file pmed.1003238.s023.pdf]

Appendix to *Evaluation of a city-wide school-located influenza vaccination program in Oakland, California with respect to vaccination coverage, school absences, and laboratory-confirmed influenza: a matched cohort study*

S1 Table. Influenza vaccines delivered by the SLIV intervention each year

Year	Schools invited to participate	Schools that participated (%)	Students eligible [a]	Students vaccinated with LAIV or IIV (%)	% of vaccinated students who received the LAIV (%)	% of vaccinated students who received the IIV (%)	Staff/teachers vaccinated
**1**	150 [b]	110 (73%)	30,045	7,518 (22%)	87% [c]	13% [c]	1,123
**2**	180 [d]	138 (77%)	35,944	10,106 (28%)	85%	15%	1,171
**3**	134	102 (76%)	34,214	7,502 (25%)	0%	100%	1,230
**4**	110	95 (86%)	34,741	7,536 (22%)	0%	100%	1,279

[a] Students eligible is the total students enrolled at the beginning of the school year in the school participating in Shoo the Flu.
[b] Included 28 pre-schools
[c] The percentage of first vaccination doses. A small number of students received more than one dose in 2014-15.
[d] Included 27 pre-schools
