# Supplementary material for: Evaluation of a city-wide school-located influenza vaccination program in Oakland, California, with respect to vaccination coverage, school absences, and laboratory-confirmed influenza: A matched cohort study
Source: PLoS Med. 2020 Aug 18;17(8):e1003238. doi: 10.1371/journal.pmed.1003238 (PMC7433855; doi:10.1371/journal.pmed.1003238)
Supplement: S2 Table — (PDF) [file pmed.1003238.s024.pdf]

Appendix to *Evaluation of a city-wide school-located influenza vaccination program in Oakland, California with respect to vaccination coverage, school absences, and laboratory-confirmed influenza: a matched cohort study*

S2 Table. Percentage of elementary students vaccinated for influenza by vaccination location in each district

Site	Vaccine type	2014-15	2015-16	2016-17	2017-18
Comparison	Doctor's office / health clinic	57.8 (54.7, 60.8)	60.5 (58.1, 62.9)	51.9 (49.2, 54.7)	49.6 (46.6, 52.6)
	School	1.1 (0.7, 1.5)	0.5 (0.2, 0.7)	0.3 (0.1, 0.4)	0.3 (0.1, 0.5)
	Other	2.9 (2.4, 3.4)	3.2 (2.5, 3.8)	3.0 (2.5, 3.5)	1.8 (1.4, 2.3)
	Error/Missing/ Don't know	2.4 (1.8, 2.9)	1.8 (1.2, 2.4)	1.2 (0.8, 1.5)	1.6 (1.1, 2.1)
Intervention	Doctor's office / health clinic	42.1 (38.5, 45.7)	41.2 (38.6, 43.9)	37.4 (35.0, 39.8)	35.5 (32.5, 38.5)
	School	13.6 (11.0, 16.2)	22.8 (19.8, 25.7)	24.2 (20.3, 28.2)	26.2 (21.5, 30.9)
	Other	0.7 (0.4, 1.0)	0.7 (0.4, 1.0)	0.5 (0.2, 0.9)	1.2 (0.8, 1.6)
	Error/Missing/ Don't know	2.8 (2.0, 3.7)	2.9 (2.0, 3.9)	1.8 (1.1, 2.5)	1.4 (0.9, 2.0)
