# Supplementary material for: Evaluation of a city-wide school-located influenza vaccination program in Oakland, California, with respect to vaccination coverage, school absences, and laboratory-confirmed influenza: A matched cohort study
Source: PLoS Med. 2020 Aug 18;17(8):e1003238. doi: 10.1371/journal.pmed.1003238 (PMC7433855; doi:10.1371/journal.pmed.1003238)
Supplement: S3 Table — (PDF) [file pmed.1003238.s025.pdf]

Appendix to *Evaluation of a city-wide school-located influenza vaccination program in Oakland, California with respect to vaccination coverage, school absences, and laboratory-confirmed influenza: a matched cohort study*

S3 Table. Percentage of elementary students whose caregiver reported they were vaccinated for influenza by vaccine type in each district

Site	Vaccine type	2014-15	2015-16	2016-17	2017-18
Comparison	Shot	48.4 (46.1, 50.8)	52.2 (50.7, 53.7)	51.5 (48.6, 54.4)	53.4 (50.7, 56.1)
Comparison	Spray	12.4 (10.3, 14.6)	11.6 (9.9, 13.3)	-- [a]	-- [a]
Comparison	Error/Missing/ Don't know	3.2 (2.6, 3.8)	2.1 (1.6, 2.6)	4.9 (4.1, 5.7)	3.0 (2.3, 3.8)
Intervention	Shot	35.8 (32.9, 38.6)	39.1 (35.5, 42.8)	52.4 (49.2, 55.6)	64.4 (59.7, 69.0)
Intervention	Spray	18.6 (14.5, 22.6)	22.5 (18.5, 26.5)	-- [a]	-- [a]
Intervention	Error/Missing/ Don't know	4.9 (3.8, 6.0)	5.9 (4.5, 7.4)	11.6 (9.2, 13.9)	3.1 (2.2, 3.9)

[a] The spray (live attenuated) vaccine was not available 2016-17 or 20.
