# Supplementary material for: Evaluation of a city-wide school-located influenza vaccination program in Oakland, California, with respect to vaccination coverage, school absences, and laboratory-confirmed influenza: A matched cohort study
Source: PLoS Med. 2020 Aug 18;17(8):e1003238. doi: 10.1371/journal.pmed.1003238 (PMC7433855; doi:10.1371/journal.pmed.1003238)
Supplement: S4 Table — (PDF) [file pmed.1003238.s026.pdf]

Appendix to *Evaluation of a city-wide school-located influenza vaccination program in Oakland, California with respect to vaccination coverage, school absences, and laboratory-confirmed influenza: a matched cohort study*

S4 Table. Difference-in-differences in cumulative incidence and total number of laboratory-confirmed influenza hospitalization per 100,000 during influenza season

	**Indirect effect**				**Overall effect**	
Season	Non-elementary age (0-4, >14 years)		Elderly (≥65 years)		All ages	
Person-years	2,493,828		324,752		2,812,600	
	Estimate (95% CI)	p-value	Estimate (95% CI)	p-value	Estimate (95% CI)	p-value
	**Difference-in-differences in cumulative incidence (95% CI)**					
2014-15	-10 (-20, 0)	0.057	-3 (-58, 51)	0.904	-8 (-17, 1)	0.084
2015-16	-5 (-12, 3)	0.210	-38 (-77, 1)	0.057	-4 (-11, 2)	0.188
2016-17	-17 (-30, -4)	0.008	-73 (-147, 1)	0.054	-15 (-26, -3)	0.011
2017-18	-37 (-54, -19)	<0.001	-160 (-267, -53)	0.004	-32 (-48, -17)	<0.001
	**Difference-in-differences in total hospitalizations (95% CI)**					
2014-15	-43 (-88, 1)	0.057	-15 (-259, 229)	0.904	-35 (-76, 5)	0.084
2015-16	-21 (-53, 12)	0.210	-171 (-347, 5)	0.057	-19 (-48, 10)	0.188
2016-17	-76 (-133, -20)	0.008	-327 (-659, 5)	0.054	-65 (-115, -15)	0.011
2017-18	-165 (-243, -86)	<0.001	-715 (-1195, -236)	0.004	-143 (-213, -74)	<0.001

Difference-in-differences compare the difference in incidence in the intervention group during a program year to the incidence in the intervention group in three pre-program years (2011-2013) to the analogous differences in the comparison group. Analyses were restricted to influenza season, defined as the period following at least two consecutive weeks in which the percentage of medical visits for influenza-like illness in California as reported by the California Department of Public Health exceeded 2.5% and prior to at least two consecutive weeks in which the percentage was less than or equal to 2.5%. Parameters were estimated using a log-linear Poisson model with an offset for population size. Standard errors and 95% confidence intervals were obtained using the delta method.
