# Supplementary material for: Evaluation of a city-wide school-located influenza vaccination program in Oakland, California, with respect to vaccination coverage, school absences, and laboratory-confirmed influenza: A matched cohort study
Source: PLoS Med. 2020 Aug 18;17(8):e1003238. doi: 10.1371/journal.pmed.1003238 (PMC7433855; doi:10.1371/journal.pmed.1003238)
Supplement: S5 Table — (PDF) [file pmed.1003238.s027.pdf]

Appendix to *Evaluation of a city-wide school-located influenza vaccination program in Oakland, California with respect to vaccination coverage, school absences, and laboratory-confirmed influenza: a matched cohort study*

S5 Table. School absence rate per 100 days and difference-in-differences in absence rate during influenza season

		School absence rate per 100 days during flu season		During flu season		During peak week of flu season (95% CI)	
Season	Student-days during flu season	Intervention	Comparison	Difference-in-difference (95% CI)	p-value	Difference-in-difference (95% CI)	p-value
**Illness-specific absences**							
2011-13 (Pre intervention)	-	2.84	2.81	-	-	-	-
2014-15	8,545,199	3.34	3.45	-0.16 (-0.54, 0.23)	0.425	-0.61 (-1.18, -0.04)	0.036
2015-16	7,355,539	3.06	3.36	-0.34 (-0.78, 0.10)	0.130	-0.27 (-0.81, 0.27)	0.335
2016-17	7,255,361	3.4	4.03	-0.63 (-1.14, -0.13)	0.014	-0.84 (-1.52, -0.15)	0.017
2017-18	7,971,492	3.29	4.06	-0.80 (-1.28, -0.31)	0.001	-2.13 (-2.87, -1.38)	<0.001
**All-cause absences**							
2011-13 (Pre intervention)	-	4.85	5.84	-	-	-	-
2014-15	8,545,199	5.56	6.36	0.15 (-0.15, 0.46)	0.326	-0.01 (-0.62, 0.60)	0.976
2015-16	7,355,539	5.24	5.97	0.24 (-0.14, 0.63)	0.216	0.31 (-0.24, 0.86)	0.264

2016-17	7,255,361	5.94	7.47	-0.47 (-0.98, 0.03)	0.067	-0.40 (-1.09, 0.28)	0.249
2017-18	7,971,492	5.77	6.74	0.04 (-0.38, 0.46)	0.842	-1.58 (-2.48, -0.68)	0.001

Each difference-in-difference estimate compares the difference in absence rates in each district in a program year compared to the three pre-program years (2011-2013), which removes any time-invariant differences between groups (measured or unmeasured). Parameters were estimated using a generalized linear model and were adjusted for month, student race, and grade. Standard errors and 95% confidence intervals account for clustering at the school level.

Note: in 2011-12, 2016-17, and 2017-18, the peak week of the percentage of influenza-like illness visits in California were the last week of December, which coincided with school breaks, so for the absentee analysis we shifted the peak week definition to closest wee k with the next highest percentage of influenza-like illness visits when both school districts were in session.
