# Supplementary material for: Evaluation of a city-wide school-located influenza vaccination program in Oakland, California, with respect to vaccination coverage, school absences, and laboratory-confirmed influenza: A matched cohort study
Source: PLoS Med. 2020 Aug 18;17(8):e1003238. doi: 10.1371/journal.pmed.1003238 (PMC7433855; doi:10.1371/journal.pmed.1003238)
Supplement: Attachment — Submitted filename: ShootheFlu_PLoSMed_Responses_reviewer_comments1.docx [file pmed.1003238.s028.docx]

Dear *PLOS Medicine* editors:

We found the peer reviewer comments to our initial submission to be positive and very constructive. My co-authors and I are pleased to submit a revised manuscript that addresses the Referees’ suggestions. We believe that this revised version is greatly improved.

---------------------------------------------------------------------------------------------------------------------**Requests from the editors:**

1. **To your data statement, please add contact information for the California Emerging Infections Program.**

Response: We have made this change in the Editorial Manager system in the section that provides additional information about data access.

2. **We ask you to adapt the title so that it is not declarative in style, and suggest: "Evaluation of a city-wide school-located influenza vaccination program in Oakland, California with respect to vaccination coverage, school absences, and laboratory-confirmed influenza: a matched cohort study".**

Response: We have made this change.

3. **We suspect that your study may best be described as a retrospective analysis of prospectively gathered data, and therefore suggest avoiding claims that the research design is "prospective".**

Response: We agree with this characterization of the study and have revised this language throughout. In addition, in the Methods section in the first paragraph under “Study design”, we state:

*“The study used a matched cohort design to evaluate the SLIV intervention program using a retrospective analysis of prospectively collected data”*

4. **To your abstract, please add some summary details from table 1 to characterize the intervention and comparison communities (e.g., median household income and ethnicity).**

Response: We have made this change.

5. **Where you quote specific findings in your abstract, such as "... the reduction in influenza hospitalizations ... was 76 ... and 165 ...", to assist readers we suggest that you adapt the text to the form "... the reduction ... was 76 individuals ...", for example, and add the size of the relevant group where appropriate.**

Response: In responding to comment #18, we revised the abstract and it no longer includes this language. Instead, we present the difference-in-differences estimates, which are easier to interpret without additional information.

6. **Where available, please add p values alongside 95% CI.**

Response: We added p-values for parameter estimates in the text of the abstract and manuscript as well as in S9 Appendix Table 2 and Table 3.

While adding p-values to S9 Appendix Table 3, we noticed that the relative reduction parameter was unstable (the bootstrap distribution of parameter estimates was non-normal). For this reason, we chose to remove the relative reduction estimates from this table. Since the primary analysis focused on difference-in-differences and the relative reduction parameter was estimated post-hoc, we consider this to be a minor change that does not impact the interpretation of our results.

7. **Please revisit your abstract to ensure that the language used (e.g., "intervention ... reduced the incidence" at line 52) is softened to reflect the observational research design (e.g., to "intervention ... was associated with a reduced incidence"), and extend this to the paper's Discussion section, for example.**

Response: We have made this change. For example, in the Discussion section the last sentences of the first paragraph now read:

*“During the first two years of SLIV, the program offered LAIV, which had low effectiveness [58,59], and on the whole, we did not observe associations between SLIV and hospitalization or absence rates in those years. In the latter two years, when the intervention delivered the IIV and the vaccine was moderately effective [41,60], we observed an association between SLIV and reduced illness-related school absences and reduced hospitalizations among age groups not targeted by SLIV, suggesting that the intervention produced herd effects.”*

8. **After the abstract, we will need to ask you to add a new and accessible "author summary" section in non-identical prose. You may find it helpful to consult one or two recent research papers published in PLOS Medicine to get a sense of the preferred style.**

Response: We have added this section.

9. **Please trim the introduction section of your paper.**

Response: We have done so.

10. **We note that you refer to a prespecified analysis plan in the methods section of your main text, and ask that you highlight any non-prespecified analyses.**

Response: We labeled any such analyses as “post-hoc”.

11. **Please adapt the early part of your discussion section so that the first paragraph consists mainly of a summary of the study's findings.**

Response: We have made this change.

12. **Please adapt journal names where needed (e.g. "PLoS ONE" for reference 69; "U S A" needs to be added to reference 42). Several references are quoted as "in press", which can probably be removed if these are "early online" versions.**

Response: We have updated the journal names and added volume/issue/pages to publications that were previously In Press and removed In Press for early online versions. We are not certain how to adapt reference 42 in the original paper, which is reference 41 in the revision, but if you provide us with additional details about what needs to be changed, we would be happy to do so.

13. **We ask you to add a completed checklist for the most appropriate reporting guideline as a supplementary document (referred to in your methods section), which may be STROBE or RECORD. In the checklist, please refer to individual items by section (e.g., "Methods") and paragraph number rather than by line or page numbers, as the latter generally change in the event of publication.**

Response: We have done so.

**Comments from the reviewers:

*** Reviewer #1:

The manuscript describes the results of a multi-year study to assess the community-wide impacts of a school-located influenza vaccination (SLIV) program that was introduced in the Oakland Unified School District (OUSD) starting in 2014.  The study uses many sources of data to estimate various effects of the SLIV program over multiple years with multiple vaccines; this approach would likely appeal to the general readership of this journal.  However, I have several major concerns with the manuscript.** 

14. **It is difficult to follow and keep track of the various endpoints, subgroups, stratified analyses, and sensitivity analyses, especially since neither the schematic of SLIV effects estimated in the study (Figure 1), nor the supplemental material (Appendix S4) include many of the analyses included in the abstract, results, and primary figures. For example, Figure 3 includes estimates among the elderly (not included in Figure 1) but does not include estimates among elementary-aged children (included in Figure 1).   The Methods section and accompanying figures should be revised for completeness and clarity.**

Response: We have updated Figure 2, which displays a schematic of different types of effects in this study, to include the outcomes measured for each type of effect. In addition, we moved the definitions of each type of effect from the supplement to the figure caption. We have also reviewed the manuscript and ensured that the terms used to refer to different types of effects are consistent.

15. **There is a lack of consistency when referring the various definitions of the influenza season considered in this manuscript that should be resolved, including:
    -       On page 11, the primary and pre-specified analysis defined the flu season starting when there were two consecutive weeks with %ILI reported to CDPH exceeding a given cutoff. In the primary manuscript, the cutoff of 2.5% was selected after comparing cutoffs of 2%, 2.5%, and 3%, as shown in Appendix S5.  However, I do not see mention of alternative cutoffs in the analysis plan.  Additionally, in Appendix S10 Fig 13, the caption suggests that the CDC flu season definition is the primary analysis.**

Response: We have revised the relevant section of the Methods section to provide more detail about our definition of influenza season and what was and was not pre-specified. The “definitions of influenza season” section now reads as follows:

*“In our pre-specified analysis plan we set the cutoff at 2%. Post-hoc, we examined seasons defined using cutoffs of 2%, 2.5%, and 3% and selected 2.5% as the primary definition because in some seasons, the 2% cutoff included weeks as early as September and as late as June, and we felt that the 2.5% cutoff best captured seasonal variation in peak influenza-like illness (S4 Appendix). We made this change only considering overall seasonal trends for influenza-like illness, before estimating any program effects.”*

Regarding Appendix S10 Fig 13, which in the revised version is Appendix S9 Fig 11, we updated the plot labeling to make it clear that the primary analysis used the CDPH definition based on the 2.5% cutoff, and the sensitivity analysis used the CDPH-based definition (from October 1 to April 30 – see response to #16 below).

16. **Is California Emerging Infection Program's influenza season from Oct 1 through April 30 (Page 11, lines 231-232) the same as the period between week 40 and week 20 of each year (Page 13, line 269; Page 14, line 290), and CDC's standard definition (as referred to in Appendix S10 Fig 13)?**

Response: We revised the caption of Appendix S10 Fig 13, which in the revised version is Appendix S9 Fig 10. Week 40 starts close to October 1^st^ and Week 20 starts close to May 15^th^. However, CEIP only collects data through April 30^th^, so we relabeled the caption and revised the relevant part of the methods section to clarify that we used the CEIP definition of flu season in the sensitivity analysis (October 1 to April 30). The absentee sensitivity analysis uses the cutoffs of week 40 and week 20 (the CDC definition of flu season), so we left that portion of the methods section as is. We chose to use the CDC definition of flu season for the absentee sensitivity analysis to be consistent with prior papers. Since CEIP did not collect data after April 30, we were not able to use the CDC definition in that sensitivity analysis.

17. **Finally, the sensitivity analysis for hospitalizations that restricted to the peak week of each influenza season is first introduced in the primary manuscript on Page 18, in the discussion of the results.  Since this was prespecified, I would have expected it to be included in the discussion of the methods (it was for absenteeism).**

Response: We have added the following sentence to the Methods section under “Statistical analysis”, “Laboratory-confirmed influenza hospitalization”:

*“We also estimated outcomes in the peak week of influenza season, defined as the week with the highest rate of influenza hospitalization in the study site.”*

18. **The analyses were designed to estimate incidence and absentee rates, but the authors sometimes report intervention effects in absolute numbers (including in the Abstract).  The total numbers aren't interpretable until they are put into a broader context and the total numbers are a simple scaling of the estimates of the rates (Page 14, line 281). I don't mind when the estimated total numbers are presented along with the estimated rates (as in Figure 3), but I am not sure how to interpret results when only the total numbers are presented. Why report only the results in total numbers when the rates are directly comparable?**

Response: We agree with the Reviewer that the absolute numbers are less generalizable than rates and more difficult to interpret on their own (though very important to the Oakland school district), so we have updated the abstract accordingly. The abstract now presents results as follows:

*“During seasons when vaccination coverage was higher among intervention schools and the vaccine was moderately effective, there was evidence of statistically significant indirect effects: the difference-in-difference (DID) in the incidence of influenza hospitalization per 100,000 in the intervention vs. comparison site was -17 (95% CI -30, -4; p = 0.008) in 2016-17 and -37 (95% CI -54, -19; p = <0.001) in 2017-18 among non-elementary school aged individuals and -73 (95% CI -147, 1; p = 0.054) in 2016-17 and -160 (95% CI -267, -53; p = 0.004) in 2017-18 among adults 65 years or older. The DID in illness-related school absences per 100 school days during influenza season was -0.63 (95% CI -1.14, -0.13; p = 0.014) in 2016-17 and -0.80 (95% CI -1.28, -0.31; p = 0.001) in 2017-18.”*

19. **There are several comparisons made, and while p-values are not presented the authors do describe results as statistically significant, or significantly different. How is statistical significance assessed throughout the manuscript? And was there any adjustment for multiple testing?**

Response: While there are multiple outcomes (influenza hospitalization, all-cause absences, illness-specific absences), and in the case of the influenza hospitalization analysis, 3 age subgroups analyzed here, they were pre-specified and thus are hypothesis driven rather than exploratory. Streiner pointed out that when a limited number of outcomes and subgroups are analyzed and they are pre-specified, it is not always productive or necessary to adjust for multiple testing. In the submitted version of the manuscript, we assessed statistical significance by determining whether the 95% confidence interval contained the null. For example, for a difference-in-difference estimate, a statistically significant result’s 95% confidence interval would not span zero. Per the PLoS Medicine Editor’s request, we have also added p-values to the manuscript.

Streiner DL. Best (but oft-forgotten) practices: the multiple problems of multiplicity—whether and how to correct for many statistical tests. *Am J Clin Nutr* 2015; 102:721-8.

20. **The claim that the school district catchment areas summarized in Table 1 are "similar" (Page 16, line 323) seems overly broad when there are noticeable differences between the two areas. The differences in median income, percentage of families below the poverty level, and a proportion of the population with college degrees could be associated with the outcomes of interest.  If these differences are indicative of systemic difference between the two areas, could these differences confound the estimates of the SLIV program effects?**

Response: We have revised the first few sentences of the Results section to highlight some of the key differences:

*“Many measured pre-intervention characteristics were similar in populations residing in the catchment areas of the intervention and comparison schools (Table 1). The median household income was lower in the intervention site than the comparison site ($51,849 vs. $61,596). Comparing the intervention site to the comparison site, the proportion Black/African American race was higher (26% vs. 17%), and the percentage of Hispanic or Latino residents was lower (26% vs. 33%). The percentage of residents with a Bachelor’s degree or higher was 15% in the intervention site and 6% in the comparison site.”*

We agree with the Reviewer that the differences between population characteristics could confound estimates of associations in this study, as is true for any observational study. The table the Reviewer referred to (Table 1) is most relevant to the associations between SLIV and influenza hospitalization since hospitalization data was collected for zip codes overlapping with each school district. For vaccination coverage and absentee outcomes, the relevant comparison table comparing districts is S2 Appendix Table 1, which shows school-level characteristics after matching. Overall, these characteristics were similar between schools, although there were also some differences in parent education and student race.

In the influenza hospitalization and absentee analyses, we adjusted for time-variant patient level confounders that were available to us. For the influenza hospitalization analysis, these included age, sex, and race. For the absentee analysis, these included student race and grade. As we point out in the manuscript in the “Statistical analysis” section under “Laboratory-confirmed influenza hospitalization”, a strength of estimating the difference-in-difference parameter is that it eliminates any time-invariant confounding and accounts for differences in pre-intervention trends between districts. Thus, for these analyses, we anticipate that using a difference-in-difference approach helped us account for confounding resulting from differences between sites that remained after matching.

21. **It looks like the Intervention/Comparison labels at the top of Appendix S3 Table 1 are flipped compared to other summaries of the two school districts, including those in Appendix S6 and Appendix 1 in the pre-specified analysis plan. Please check the column labels.  Additionally, based on the description of the matching algorithm on starting on Page 8, line 159, I was expecting Appendix S3 Table 1 to also include summaries of school-level percentages of English language learners and students receiving free lunches.**

Response: Thank you very much for catching the mislabeling of the columns in Appendix S3 Table 1 (S2 Appendix Table 1 in the revision). We have corrected it. We have also added rows to this table for the school-level percentages of English language learners and students receiving free lunches.

22. **Why are 95% CIs not reported along with vaccination coverage estimates (pages 16-17, Appendix S6)? Since they are reported in the Abstract and described in the Statistical Analysis section (Page 12, line 235), they should be reported throughout the manuscript.**

Response: We have added the 95% confidence intervals for the difference in vaccination coverage estimates to the second paragraph of the Results section as well as to S9 Appendix Table 2 and 3. However, we did not add these to S5 Appendix Fig 1 in the revised version because the available data for the pre-intervention school district data is aggregated and would not allow us to calculate 95% confidence intervals for each district that account for clustering within schools. While we could include a supplementary table with the confidence intervals for vaccination coverage by parent education level in our survey, we chose not to because in response to comment #50 we decided to present these in a separate manuscript.

23. **The weekly time series in Fig 2 lets us assess whether the trends in the incidence of flu-hospitalization are parallel within a given flu season, but the analyses are done comparing annual flu seasons (so cumulative incidence for the 2015-16 season). How do the weekly (or daily in the case of absenteeism) time series allow us to assess how reasonable the parallel trend assumption is for the DID analyses that are done?  And are there similar assessments for the stratified analyses?  Lastly, why were school absence trends used to assess whether the equal trend assumption was met for the analyses of influenza hospitalizations (Page 13, line 257)?**

Response: Examining time series prior to an intervention is the standard approach for assessing the parallel trends assumption. Examining cumulative incidence prior to the intervention may average over important differences in pre-intervention trends between intervention and control that should be taken into account. We have added figures showing the pre-intervention and intervention period weekly incidence of influenza hospitalization for non-elementary aged individuals and elderly individuals since these subgroups were included in our primary analysis in the pre-analysis plan for this study. These are located in S6 Appendix, and they show that pre-intervention trends were similar in these age groups.

We added the following sentence to the “statistical analysis” section under “laboratory-confirmed influenza hospitalization”:

*“Examination of pre-intervention influenza hospitalization trends indicated that the equal trends assumption was met (Fig 3, S6 Appendix Figs 1-2).”*

And we added the following sentence to the “statistical analysis” section under “school absence rates”:

*“Examination of pre-intervention absence trends indicated that the equal trends assumption required for valid DID analysis was met (S6 Appendix Figs 3-4).”*

24. **Based on the negative control analysis for absenteeism, the authors conclude that differential measurement error may impact the primary analysis (Page 22, line 454).  The misclassification analysis gets a lot of attention, but the results of negative control analysis could be explained by many other possible sources of unmeasured confounding. Why was misclassification the only source considered?  Could the differences seen in Table 1, along with the results of the negative control analysis be indicative of a poor match for the comparison district?  Or could the demographics have shifted differentially in the two districts?**

Response: We agree with the Reviewer that unmeasured confounding may have influenced our estimates, as is possible in all observational studies. The reason we chose to focus the probabilistic bias correction analysis on misclassification is that through conversations with colleagues at each school district, we were able to assign reasonable distributions of sensitivity and specificity to the classification of each type of absence. For unmeasured confounders, such as those with discrepancies in Table 1 (e.g., median household income), such an analysis would require us to guess the magnitude of association between each unmeasured confounder and the outcome (e.g., the relative risk). Unfortunately, because we do not have any data in this study to estimate the magnitude of association, a probabilistic analysis to correct for unmeasured confounding would be based mostly on conjecture which we felt would be less valuable.

**Minor comments:**

25. **In Table 1, how are the 95% CI's computed for the Median household income?**

Response: the American Community Survey provided an estimate for median household income and the margin of error for each estimate. We followed the instructions for calculating a 95% confidence interval in the ACS documentation. The formula is:

Lower bound: median estimate - (MOE / 1.645)*1.96

Upper bound: median estimate + (MOE / 1.645)*1.96

Where MOE is the margin of error for a 90% confidence interval, and MOE/1.645 is equal to the standard error of the median estimate.

<https://www.census.gov/content/dam/Census/programs-surveys/acs/guidance/training-presentations/20180418_MOE.pdf>

26. **One school dropped out of the vaccine coverage survey, how is that school and its matched pair accounted for in the participant flow in Appendix S9?  Where there originally 45 or 46 schools?**

Response: We have updated this figure (now S8 Appendix) to show the number of schools included in each dataset in each survey. The 2016-17 survey included data from 21 schools in OUSD and 22 in WCCUSD. The 2017-18 survey included data from 22 schools from each district. We also revised the following sentence in the Methods section under “Outcomes and data sources”, “Vaccine coverage survey”

*“We distributed the surveys in 22 of the 34 matched school pairs (22 K-5 schools in the intervention district and 22 K-6 schools in the comparison district).”*

27. **The categories for the analysis of vaccine coverage by caregiver education level (Appendix S10, Figure 4) and Page 17, line 352 should match than those reported earlier in Appendix S6 and be discussed consistently in the manuscript.**

Response: based on Reviewer 3 feedback (Comment #50) we have removed that sentence from the manuscript and will report subgroup analyses in a separate manuscript to simplify the reporting for the primary results. This comment is no longer applicable to the revised manuscript.

28. **Do the estimates in Figure 2 include ICU admissions? Are these total numbers, standardized for potential differences in age groups across the two areas?  I would also expect to see these trends for the age subgroups used to obtain the estimates in Figure 3.**

Response: We have updated the caption for this figure (Fig 3 in the revised version) to indicate that influenza hospitalization includes intensive care unit admission. We have also added age-stratified figures to S6 Appendix, Figs 1-2. These plots display the weekly incidence, which is calculated as the total number of laboratory-confirmed influenza hospitalizations, including intensive care unit admissions, divided by the population in that site. Our pre-analysis plan did not call for age standardization of incidence rates. Below, we show both Fig 3 and the same plot with age standardized rates using the age distribution of the intervention site as the standard. The plots are very similar because the age distributions between sites were very similar during the study period. Because the plots are so similar and we did not pre-specify age standardization, we have chosen to include the plot that does not standardize by age in the manuscript.

This figure caption now reads:

***“Fig 3. Weekly incidence of inpatient laboratory-confirmed influenza prior to and during the intervention among all ages***

*Weekly incidence proportion of laboratory-confirmed influenza hospitalizations including intensive care unit admissions between Week 40-52 and Week 1-20 of each year. Hospitalizations included school district residents tested at health care facility laboratories located in zip codes overlapping with OUSD and WCCUSD (Alameda County Public Health Department, Children’s Hospital Oakland, Contra Costa Public Health Department, Kaiser Permanente, Sutter Health). Population denominators were obtained from the U.S. 2010 Census using the same set of zip codes.”*

**No age standardization**

**Age standardized by intervention site age distribution**

29. **How was illness-specific absence defined?**

Response: We have added the following to the Methods section under “Outcomes and data sources” and “School absence records”:

*“Absences were classified as related to illness or not based on parent report by each school district.”*

30. **Does the school-absenteeism analysis use only the matched schools in each district, or all schools in both districts?**

Response: This is addressed in the final sentence of the “Study Design” section in the Methods section:

*“To measure impacts on school absences we pre-specified inclusion of all public elementary schools enrolling Kindergarten to grade 5 (“K-5”) (50 intervention schools, 34 comparison schools) rather than the matched subset used to design the vaccine coverage survey in order to maximize precision.”*

31. **The caption for Figure 4 should be edited to reflect the definition of flu season that is used.**

Response: We have added this.

32. **What are the adjusted and unadjusted analyses presented in Appendix S10 Figure 14?  And the accompanying caption should clearly define the flu season definitions.**

Response: We have updated the caption to S9 Appendix Fig 12 in the revised version as follows:

*“Parameters were estimated using a generalized linear model. The left-hand panels (“Unadjusted”) include results for unadjusted models that did not adjust for month, student race, and grade. The right-hand panels (“Adjusted”) include results for models that adjusted for month, student race, and grade. Standard errors and 95% confidence intervals account for clustering at the school level.”*

33. **The pre-specified plan described the study as a retrospective matched cohort study, but the manuscript refers to the study as prospective. Can you please clarify?**

Response: Please see our response to comment #3.

34. **How were the estimates of vaccine effectiveness (page 7 and Appendix S2) obtained?**

Response: as indicated in the caption to Fig 1 in the revised manuscript, these estimates were obtained from the U.S. Influenza Vaccine Effectiveness Network. In addition, the sentence in the main text referencing Fig 1 includes these citations:

39.	Grohskopf LA. Prevention and Control of Seasonal Influenza with Vaccines. MMWR Recomm Rep. 2016;65. doi:10.15585/mmwr.rr6505a1

40. Centers for Disease Control. Seasonal Influenza Vaccine Effectiveness, 2016-2017. 9 Apr 2019 [cited 14 Jun 2019]. Available: <https://www.cdc.gov/flu/vaccines-work/2016-2017.html>

41.	Rolfes MA, Flannery B, Chung JR, O’Halloran A, Garg S, Belongia EA, et al. Effects of Influenza Vaccination in the United States During the 2017–2018 Influenza Season. Clin Infect Dis. 2019;In Press. doi:10.1093/cid/ciz075

35. **The manuscript described power calculations for the vaccine coverage survey but similar computations for the laboratory-confirmed influenza hospitalizations and school absenteeism endpoints (those can be found in the pre-specified analysis plan). These should be organized better.**

Response: given that this manuscript is already quite lengthy, as pointed out by multiple Reviewers, we have chosen to add a supplement with the power calculations for each endpoint. We moved the power calculation for the vaccine coverage survey to this supplement and moved the power calculations for influenza hospitalization and school absenteeism from the pre-analysis plan there as well. This content is in S3 Appendix.

36. **I could not find a pre-specified plan for the matching of cohorts or for of the vaccine coverage survey.**

Response: The Reviewer is correct that these were not included in the pre-analysis plan. Regarding matching schools, we did pre-specify in the proposal to our funder and in our Institutional Review Board protocol for the vaccine coverage survey that we would use the genetic matching algorithm to match K-5 elementary schools using pre-intervention variables from the California Department of Education. These documents listed the variables that we used for matching and provided details about the matching, and we can provide them upon request. The comparison district (West Contra Costa Unified) was not pre-specified because in addition to the scientific process of identifying potential school districts that had similar characteristics to Oakland Unified, we went through a lengthy process of reaching out to local school districts and assessing their willingness to participate in the study.

Regarding the vaccine coverage survey, we originally planned a much more extensive survey involving prospective repeated measurements. However, after two years of piloting, we did not enroll sufficient participants to justify that approach. For this reason, we chose to conduct a one-page, anonymous survey at a single time point to measure influenza vaccine coverage in 2017, and since that approach was more successful, we repeated the process the following year. We did not pre-specify the analysis of the vaccine coverage survey data since this analysis was relatively straightforward and did not include more complex aspects of other analyses, such as covariate adjustment or difference-in-difference models.

37. **I could not find the code to replicate the analyses online.**

Response: The Open Science Framework page embeds three Github repositories associated with this analysis: <https://osf.io/c8xuq/> There are separate repositories for analyses of vaccine coverage, influenza hospitalization, and school absences.
 ***** Reviewer #2:

In this interesting study, the authors evaluated the impact of school-located influenza vaccination (SLIV) programs implemented in >95 preschools and elementary schools in Oakland, CA during the 2014-15 to 2017-18 influenza seasons. The authors used multiple data sources to examine vaccine coverage, school absences, and laboratory-confirmed influenza hospitalizations. They used a matched cohort design with a comparison community, and used a difference-in-differences (DID) approach to the analyses for hospitalization incidence and absenteeism.

I was impressed by: 1) the inclusion of multiple outcomes determined from independent data sources; 2) the rigor and comprehensiveness of the analyses conducted (with numerous sensitivity and subgroup analyses), and how the authors addressed a number of challenges along the way; 3) the interesting findings of the effects of the program against relatively specific outcomes when differences in vaccine coverage were observed and the vaccine was effective; 4) the appropriate interpretation of the results and the fulsome discussion; and 5) the quality of the writing.

I believe this manuscript would be suitable for publication with just a few minor changes:**

38. **Abstract Line 30: I believe the surveys should be labelled as 2017 and 2018 rather than 2016 and 2017.**

Response: Thank you for pointing this out. We have made this correction.

39. **Abstract, Lines 45-49: I am wondering if it would be better to report the reductions in outcomes as rates rather than absolute counts. I can appreciate the desire to report the reductions as absolute counts, but would like to hear the authors' rationale for choosing counts over rates.**

Response: We agree. Please see our response to #18.

40. **S10 Appendix Table 1: DID in total hospitalizations, Elderly, 2016-17: should be -5 rather than 5. In later rows, the authors should use 2014-15, 2015-16, etc. for the row headings, instead of 1415, 1516, etc. for consistency.**

Response: We have corrected the season labels in the table. As for the relative reduction estimate for the elderly, the value 5 (-117, 59) is correct. Negative values are not possible for relative reductions since these are defined as 1 – RR x 100%. For this particular result, the RR = 0.95. The reason the lower bound of the confidence interval is negative is because the standard error for this estimate was very large as a result of the small number of hospitalizations in this age group in 2014-15.

41. **Line 406: should be S10 Supplement, not S11 Supplement**

Response: We have made this correction.

**Jeff Kwong

*** Reviewer #3:

GENERAL COMMENTS
This is a very nicely designed study, using aa matched prospective cohort design (matching school districts) and a DID analysis to evaluate the impact on school-located influenza vaccination (SLIV) on:   (a) influenza vaccination rates of schoolchildren, (b) school absenteeism, (c) indirect effects (child and adult lab-confirmed hospitalization rates). The study found that during the first two years of SLIV, self-reported vaccination coverage was not higher in intervention schools (when 22-28% of children were vaccinated in school) and flu VE was suboptimal, but in the latter two years (2016/17 and 2017/18) it was higher and there was also a DID difference in the expected direction for school absenteeism and pediatric and adult hospitalizations (same % of children receiving SLIV, but these 2 years the vaccine was more effective).

The study is novel in that it is methodologically rigorous yet also measures school absenteeism and hospitalizations as indirect outcomes (most prior RCTs of SLIV have not).The study is limited by inherent weaknesses of the design (namely lack of an RCT, self-reported influenza vaccinations); however given the fact that in today's world it is quite unusual to be able to randomize schools and to conduct such large studies within an RCT, the study and findings are important. The authors do a nice job describing the limitations.

The findings are complicated by multiple co-occurring events or findings—across the 4 years, when similar numbers of students received SLIV vaccinations:
*       Overall influenza vaccination rates were lower in intervention schools in Year 1, no different in Year 2, but then much higher in years 3-4 but almost entirely due to vaccination rates in control school districts dropping precipitously rather than vaccination rates rising in intervention schools presumably due to SLIV. This suggests the possibility that it was not SLIV that caused these findings, but rather some unknown drop in vaccination rates in the control school district.
*       LAIV (which was given in >85% of SLIV cases) was minimally effective in year 1, not effective in year 2, but IIV which was given exclusively in years 3-4 was effective those years based on national data. So the study findings would predict no impact in years 1-2, but an impact in years 3-4 based merely on national VE data—but only if vaccination rates are indeed higher in intervention vs control school districts.
All of this makes it challenging to follow and understand the findings; but having said that the paper is extremely well written given these constraints.**

42. **One key problem I see is self-reported vaccination rates, and particularly a single survey to assess flu vaccinations received over 3 years—the accuracy of this is unclear. Why was CA immunization registry data not assessed to at least verify accuracy? It is possible that recall is better for SLIV districts because parents needed to give consent for vaccination—causing a potential positive (not conservative) bias in ascertainment of vaccination rates. Also the low response rates for the parent surveys, and much lower response rates for the intervention schools (28% vs 38% in control schools) makes these findings a bit more suspect, though I don't have apriori thoughts about the possible direction of bias.**

Response: We agree that the use of self-reported influenza vaccination data is a limitation of this study. In paragraph 10 of the Discussion section, we discuss this issue and also mention the low response rates to our survey.

We thoroughly assessed whether it was possible to use California immunization registry data and had lengthy conversations with county and state level public health department staff to explore what was possible. Unfortunately, following these conversations we concluded that it would not be possible to validly compare coverage estimates from our study to those from the California immunization registry. Our primary concern was that, in part because California does not mandate providers to report vaccinations to the registry, vaccination records are incomplete, and vaccination rates calculated from the registry are much lower than those from other sources, such as the CDC’s Behavioral Risk Factor Surveillance System, which obtains annual estimates of influenza vaccination coverage through self-report.

Regarding the registry, we have added the following to paragraph 10 of the Discussion section:

*“It was not possible to validly compare our results to coverage estimates from the California Immunization Registry data because prior to and during the SLIV intervention, many local vaccine providers did not consistently enter data into the registry as there is no mandate to do so in California.”*

43. **This is an enormous manuscript; difficult to wade through. I would suggest eliminating race/ethnicity comparisons throughout to make it a bit less cumbersome. I don't see apriori hypotheses for race/ethnicity, and it is confusing since vaccine uptake is lower among black patients and samples get small for the surveys.**

    Response: We agree with the Reviewer and have removed subgroup analyses for race/ethnicity, parent education level, and grade from this manuscript. We will prepare a separate manuscript to report those results. **SPECIFIC COMMENTS
    INTRODUCTION
    This section is well written and includes many SLIV-related publications. It also lays the rationale well for this current study- namely that the prior RCTs tended to not study school absences or flu-related illnesses, and the prior studies that did evaluate flu-related hospitalizations or illnesses had somewhat conflicting findings.


    METHODS
    SLIV intervention:
    This section is well written.

    Influenza VE:**
44. **Although space is of concern, since so much of the findings depend on the national vaccine effectiveness, I suggest either adding the table to the main article (rather than appendix) or stating numbers in the text—eg VE for SLIV in Years 1-2, and for IIV in years 3-4.**

Response: We agree that information about vaccine effectiveness is important for contextualizing our findings, so we have moved what was originally S2 Appendix to the main text. It is now Figure 1, and all other figures have been renumbered.  **Study Design:
The matching of intervention vs control school districts (cities) was done carefully.**

45. **The vaccine coverage survey is problematic since there was one survey that asked parents to recall influenza vaccinations for the prior 3 years (2014-15, 2015-16, 2016-17 flu vaccination seasons), and a second survey that asked about 2017-2018. While prior studies have found good recollection of parental reports of flu vaccinations received in the past vaccination season, I am unsure how accurate self-report would be for 3 seasons.  Having said that, there may not be bias in accuracy between intervention and control schools, but there may be bias if the SLIV process (and consent for SLIV) spurred recall of vaccinations received. This is particularly important because the entire study seems to rest upon a DROP in vaccination rates in the control city (school district)- for unclear reasons.**

Response: We agree with the reviewer that parental recall of influenza vaccination may be inaccurate and that it is possible that the presence of the SLIV intervention affected recall. We have added the following text to paragraph 9 in the Discussion section to more thoroughly address the limitations of caregiver recall (new additions are in bold):

*“Coverage estimates for 2014-15 and 2015-16 may be more vulnerable to measurement error because they rely on a 2-3 year recall period. Overall, our vaccination coverage estimates were consistent with caregiver-reported national and California-specific estimates from the Centers for Disease Control and Prevention [12,74].* ***Nevertheless, it is possible that caregiver recall in the intervention district differed from that in the comparison district; the presence of the SLIV intervention may have increased parents’ likelihood of accurately recalling their child’s vaccination status.****”*

We would like to clarify that we suspect that the lower influenza vaccination coverage in the comparison site in Year 3 (2016-17) may reflect the switch from the LAIV (nasal spray vaccine) being available in 2014-15 and 2015-16 to only the IIV (injectable vaccine) being available in 2016-17 and 2017-18 due to concerns about LAIV effectiveness. We address this in the third paragraph of the Discussion section—new additions to this section to address this comment are indicated in bold:

*“In addition, the switch from LAIV to IIV in the third year of the Shoo the Flu intervention may have inhibited larger increases in coverage because many children and/or caregivers prefer the nasal spray generally and in a school setting, and media coverage of poor LAIV effectiveness may have increased vaccine hesitancy for all influenza vaccine formulations.* ***In the comparison site, the 10% reduction in vaccination coverage between 2015-16 and 2016-17, when LAIV was discontinued, was on par with the approximately 12% of students vaccinated by LAIV in 2014-15 and 2015-16 in that site (S9 Appendix Fig 3, Table 2).*** *Yet, vaccination coverage in the intervention district grew relative to the comparison district over time, in part because the comparison district coverage did not recover from the decline associated with the switch from LAIV to IIV vaccines. It is possible that coverage in the intervention district vaccine coverage will continue to grow in future years as the intervention builds trust and recognition.”*

46. **The analytic approach is otherwise fine. Of note, I did not understand in the method section what differential ascertainment of school absences (measurement error) meant.**

Response: We have added the following sentence to the Methods section under “Statistical Analyses” and “School absence rates”:

*“Differential measurement error could have occurred if absences were recorded with different levels of accuracy between the intervention and comparison districts.”*
 **RESULTS
 Influenza vaccination coverage:**

47. **This section is mostly fine, except I would point out that the difference in coverage between SLIV and control districts was almost entirely due to a DROP in coverage in the control district rather than a rise in coverage in SLIV schools. This lack of a rise in SLIV schools suggests that most SLIV vaccinations were simply substituting for vaccinations otherwise given in doctor's offices. It also brings up the possibility that something unusual happened in the control city to account for the drop in vaccination rates and that the ultimate findings might not be due to SLIV.**

Response: Please see our response to #45.

48. **Influenza Hospitalization
    Hospitalization rates actually ROSE in both cities during the time of a moderately effective vaccine, and the major reason for the DID differences is that they rose markedly in the comparison city in 2016-2017 and 2017-2018 (this is also perhaps related to the marked drop in influenza vaccination rates in that city).**   

    Response: In California, the influenza epidemics in 2016-17 and particularly 2017-18 were substantially larger than those in 2014-15 and 2015-16 – see here:

<https://www.cdph.ca.gov/Programs/CID/DCDC/CDPH%20Document%20Library/Immunization/Annual2016-2017.pdf>

<https://www.cdph.ca.gov/Programs/CID/DCDC/CDPH%20Document%20Library/Immunization/Annual2018-19.pdf>

As the reviewer points out, if influenza vaccination rates were lower overall in the comparison site than the intervention site in 2016-17 and 2017-18, it may have contributed to higher influenza hospitalization rates. While we cannot definitively attribute the lower hospitalization rates in the intervention site to the SLIV intervention in 2016-17 and 2017-18 because we do not have data on influenza vaccination coverage in our two sites for all age groups, our results suggest that the intervention may have produced herd effects.

49. **Absenteeism: I would add the pre-intervention data to Figure 4, rather than keeping it only in the appendix.**

Response: Figure 4 contains difference-in-difference estimates, which take the difference in absence rates in the intervention site during vs. prior to the intervention and subtract the difference in rates in the comparison site during vs. prior to the intervention. Thus, this figure already implicitly includes pre-intervention data.

50. **One suggestion is to delete race/ethnicity subgroup analyses in this very extensive manuscript. There is some benefit to keeping race/ethnicity but it could be in a separate manuscript.**

Response: We agree with the Reviewer and have removed subgroup analyses for race/ethnicity, parent education level, and grade from this manuscript. We will prepare a separate manuscript containing those results.

51. **I do not follow the outcome misclassification section when only reading the text. I understand there was a peculiar decrease in illness-specific school absences outside of the flu season, and this is problematic, but the explanation was not sufficient for me to understand what was actually done "under assumed distributions of sensitivity and specificity" or how that reassured the authors that this did not influence the findings sufficiently.**

    Response: We have added the following text (new additions in bold) to the Methods section under “Statistical analysis” and “School absence rates” to provide more detail about how the probabilistic bias analysis was performed:

*“As we describe in the Results, the negative control analysis indicated possible differential measurement error of school absences, so we performed a post-hoc probabilistic bias analysis to quantify the possible influence of outcome misclassification on our results [52]. In this analysis, we assumed a range of possible values of the sensitivity and specificity of absence classification based on conversations with data managers in each school district (S8 Appendix Fig 1). For example, in the comparison district, we assumed that the sensitivity of all-cause absences (the probability that a true absence of any cause was classified as such by the school district) that the sensitivity was most likely to be close to 1 and in very few instances was less than 0.50. In a simulation, we drew from the distributions of sensitivity and specificity to calculate the bias-corrected absence rate correcting for possible misclassification under a range of plausible scenarios; we repeated this process 1,000 times to obtain distributions of bias-corrected difference-in-difference estimates (S8 Appendix).”*
 **DISCUSSION**

52. **In paragraph 2 the authors state that the vaccination rates for the intervention city was higher than rates for the control city because the latter "did not recover from the decline associated with the switch from LAIV to IIV vaccines." This might be true, but nationally vaccination rates did not drop more than 1-2 percentage points during this time, and the authors do not show data to substantiate this claim. For example, how much LAIV was used in the control city prior to its termination? Is this all speculation or are there data? Does CAIR provide any answers—ie what was the city-wide vaccination rates in CAIR before and after this time period? I am worried that something else, unmeasured, may be going on.**

Response: We added S9 Appendix Figure 3 and Table 2, which show the proportion of elementary school students receiving IIV vs. LAIV each season by district as reported by caregivers in our vaccine coverage survey. In the comparison site, in 2014-15 and 2015-16 approximately 12% of elementary school students were vaccinated with the LAIV. The drop in overall coverage from 2015-16 to 2016-17 in the comparison site was 10%. These results support our hypothesis that the SLIV intervention helped maintain higher vaccination coverage in spite of the lack of availability of LAIV in 2016-17 and 2017-18. That being said, as in any observational study, we cannot fully rule out the possibility of unmeasured confounding.

We have added the following text to the Results section in paragraph 3:

*“In 2014-15 and 2015-16, seasons when either IIV or LAIV were available, 48-52% of students received the IIV and 12% of students received the LAIV in the comparison site, and 36-39% of students received the IIV and 19-23% of students received the LAIV in the intervention site (S9 Appendix Fig 3, S9 Appendix Table 2).”*

We have added the following text to paragraph 3 of the Discussion:

*“In addition, in the comparison site, the 10% reduction in vaccination coverage between 2015-16 and 2016-17, when LAIV was discontinued, was on par with the approximately 12% of students vaccinated by LAIV in 2014-15 and 2015-16 (S9 Appendix Fig 3, S9 Appendix Table 2).”*

Regarding CAIR, please see our response to comment #42.

53. **The paragraph on potential substitution is fine, but perhaps over-calling the impact of SLIV, since it implies that without SLIV the coverage rate in the intervention city of Oakland would have dropped markedly. This did not occur nationally (flu vaccination rates for 5-12yr olds dropped by 2 percentage points between 2015/16 and 2016/17) or I believe statewide across California—so why would one expect it to drop so much?**

    Response: While we cannot know with certainty what the vaccination coverage level would have been in the intervention site if LAIV had been offered in 2016-17, if we assume that the comparison site is a reasonable counterfactual, then the pattern of coverage that we observed in the comparison site may reflect what would have happened in the absence of the SLIV intervention. To more clearly highlight this limitation, we have revised language of this paragraph, which now reads:

*“Our findings suggest that both phenomena may have occurred in this study. Coverage was higher in the intervention versus comparison district in the final two years of the evaluation, suggesting that some otherwise unvaccinated children were vaccinated by the SLIV intervention. In addition, the proportion of students vaccinated at school in the intervention district (24-26% in the latter two years (S9 Appendix Fig 2)) exceeded the difference in coverage between districts (7-11%). While we cannot definitely assess what the vaccination coverage trends would have been in the intervention site in the absence of the SLIV intervention, these findings suggest that approximately 15% of students vaccinated by the SLIV intervention may otherwise have been vaccinated through other means.”*

**Overall the rest of the discussion section was well done.**
